# Persistent effects of pre-differentiation manganese exposure on adipogenesis and metabolic function in differentiated 3T3-L1 adipocyte cells

**DOI:** 10.1016/j.hazadv.2026.101506

**Published:** 2026-08-22

**Authors:** Rehan Malhi, Maximus Wong, Varda Qudratullah, Daneen Gondal, Alexey A. Tinkov, Anatoly V. Skalny, Aaron B. Bowman, Michael Aschner, Beatriz Ferrer

**Affiliations:** a Department of Molecular Pharmacology, Albert Einstein College of Medicine, 1300 Morris Park Avenue, 10461, Bronx, NY, United States; b Laboratory of Molecular Dietetics, IM Sechenov First Moscow State Medical University, Moscow, Russia; c Laboratory of Ecobiomonitoring and Quality Control, Yaroslavl State University, Yaroslavl, Russia; d School of Health Sciences, Purdue University, West Lafayette, IN, 47907, United States

**Keywords:** Manganese, Adipogenesis, Liipid metabolism, Antioxidant defense

## Abstract

Obesity is a multifactorial metabolic disorder influenced by genetic, lifestyle, and environmental factors. Increasing evidence suggests that early-life environmental exposures can induce persistent alterations in adipose tissue, contributing to long-term metabolic dysfunction. Manganese (Mn) is an essential trace element involved in mitochondrial function, redox homeostasis, and energy metabolism; however, both Mn deficiency and overexposure have been associated with adverse metabolic outcomes. Despite epidemiological studies linking Mn exposure to obesity-related disorders, the cellular mechanisms underlying Mn-mediated metabolic disruption remain poorly understood. Here, we investigated whether transient Mn exposure during the early adipogenesis induces persistent molecular and metabolic alterations in mature adipocytes. Using the 3T3-L1 cell line, pre-adipocytes were exposed to increasing Mn concentrations (0, 5, 10, 50, 100, or 500 μM) during the first 48h of differentiation (confluence phase). Mn was subsequently removed, and cells were either maintained as non-differentiated controls or differentiated using a defined 3T3-L1 protocol designed to generate adipocytes with white morphology and beige-like features. Upon maturation, adipogenic markers, lipid accumulation, β-adrenergic-induced lipolysis, glucose transporter gene expression, and antioxidant defense-related protein and gene expression were assessed. Early Mn exposure elicited persistent concentration-dependent alterations in adipogenic programming, characterized by reduced lipid accumulation and sustained suppression of the adipogenic regulators PPARγ and C/EBPα. Fully differentiated adipocytes also exhibited persistently impaired expression of glucose transporter genes and reduced hormone-sensitive lipase activation, leading to diminished lipid mobilization under β-adrenergic stimulation. Combined, these findings demonstrate that transient Mn exposure during the initial stages of adipocyte differentiation is sufficient to cause long-term alteration of adipogenic programming and provides evidence for a mechanism of persistent metabolic disruption. Importantly, lower Mn concentrations were associated with selected persistent alterations in adipocyte maturation, whereas the strongest effects observed at 500 μM Mn likely included cellular stress or toxicity-associated responses.

## 1. Introduction

Obesity is a complex, multifactorial disease characterized by excessive or abnormal fat accumulation, especially in adipose tissue. Excessive lipid accumulation and impaired adipocyte differentiation lead to an increase in oxidative stress and chronic low-grade inflammation, which increase the risk of type 2 diabetes, cardiovascular disease, and other metabolic disorders (Chait and den Hartigh, 2020; Olivares-Vicente and Herranz-López, 2025). While factors such as high-calorie diets, sedentary lifestyles, and genetic susceptibility are well established contributors to obesity, growing evidence supports a role for environmental exposures in modulating metabolic health (Lubrano, Genovesi et al., 2013; Heindel, Howard et al., 2022; Sen, Fan et al., 2024). Among these, trace elements have gained attention as potential endocrine and metabolic disruptors, capable of influencing energy homeostasis, adipose tissue function, and systemic metabolism.

Manganese (Mn) is an essential trace element required for numerous physiological processes, including regulation of the nervous system, antioxidant defenses, immune function, energy metabolism, and glucose and lipid metabolism (Li and Yang, 2018; Baj, Flieger et al., 2023). Mn is primarily obtained through the diet and drinking water and serves as a cofactor for several enzymes critical to mitochondrial and metabolic functions. Mn is required for optimal enzymatic function, manganese superoxide dismutase (SOD2), a key enzyme in mitochondrial redox homeostasis (Luk, Yang et al., 2005; Culotta, Yang et al., 2006), and pyruvate carboxylase, which catalyzes the conversion of pyruvate to oxaloacetate, thereby supporting tricarboxylic acid cycle activity and cellular energy metabolism (Baly, Keen et al., 1985; Baly, Keen et al., 1986). Despite its essential role, Mn exhibits a narrow physiological range. While low concentrations appear to be protective (Pu, 2024), both Mn deficiency and overexposure have been reported to be detrimental (Kwiecinski and Nowak, 2009; Ganeshan, Sainath et al., 2011; Shan, Chen et al., 2016; Kang, Lee et al., 2023; Hu, Tang et al., 2025).

Environmental and occupational Mn overexposure has been shown to induce oxidative stress, impair mitochondrial function, and promote cellular dysfunction. Further, Mn has reported insulin mimetic effects (Baly, Curry et al., 1984; Subasinghe, Greenbaum et al., 1985; Baly, Keen et al., 1986; Baly, Schneiderman et al., 1990). Consistent with this, *in vitro* experiments demonstrated that Mn exposure enhanced glucose uptake in a neuronal model of Huntington’s disease, suggesting interactions with insulin-related signaling pathways (Bryan, Nordham et al., 2020). From a clinical perspective, epidemiological studies further support that both Mn deficiency and excess are associated with the development of type 2 diabetes (Shan, Chen et al., 2016).

Recent evidence has further linked altered Mn levels to obesity and compromised metabolic health, although these findings remain inconsistent. In adults, higher Mn levels have been correlated with increased visceral adiposity and altered body fat distribution (Tao, Huang et al., 2022). In contrast, studies in children and adolescents reported both positive and negative correlations between Mn levels and obesity-related outcomes (Fan, Zhang et al., 2017; Gonzalez-Dominguez, Millan-Martinez et al., 2022; Ngu, Skalny et al., 2022; Tao, Huang et al., 2022; González-Domínguez, Domínguez-Riscart et al., 2023). Notably, elevated cord-blood Mn concentrations have been positively associated with an increased risk developing obesity later in life, suggesting that early-life Mn exposure may have lasting metabolic consequences (Yu, Cao et al., 2013; Dai, Zhang et al., 2021).

Increasing evidence supports the concept that early-life environmental stressors, such as nutritional imbalances and chemical exposures, can contribute to the development of metabolic diseases including obesity and cardiovascular diseases in adulthood (Oestreich and Moley, 2017; Maguolo, Gabbianelli et al., 2023). Adipocyte differentiation is a tightly regulated process that is highly sensitive to metabolic and environmental stressors (Heindel, Howard et al., 2022). Disruption of adipogenic programming during critical windows of development may lead to long-lasting changes in adipocyte function, thereby predisposing individuals to obesity and metabolic disorders.

Although Mn is a well-established neurotoxicant, its potential role as a metabolic disruptor during early developmental stages remains less understood. The 3T3-L1 cell line, derived from mouse embryonic fibroblast, is widely used to investigate the mechanisms underlying obesity, diabetes, and associated metabolic diseases (Pereira-Fernandes, Vanparys et al., 2014; Stel and Legler, 2015; Boudalia, Belloir et al., 2017). Exposure of 3T3-L1 pre-adipocytes to exogenous agents during the initial stages of differentiation provides an *in vitro* model to assess the long-term consequences of early-life environmental insults on adipogenic programming. In this study, we investigated whether transient Mn exposure during the early stages of differentiation, specifically the 48-hour post-confluence period before hormonal induction, induces persistent molecular and metabolic alterations in fully differentiated adipocytes. This approach enables evaluation of whether early Mn-induced disruptions are retained after differentiation, with potential implications for lipid metabolism, mitochondrial function, and adipocyte phenotype, thereby providing mechanistic insights into how early environmental exposures may contribute to long-term metabolic dysfunction.

## 2. Materials and methods

### 2.1. Cell culture

3T3-L1 pre-adipocytes obtained from the American Type Culture Collection (ATCC, USA, Cat. No CL-173, RRID:CVCL_0123) were maintained in pre-adipocyte medium, containing Dulbecco’s Modified Eagle Medium (DMEM, Gibco, USA, Cat. No 11995–040), supplemented with 10% Newborn Calf Serum (NBCS, Gibco, New Zealand, Cat. No 26010–074) and 1% penicillin-streptomycin (Gibco, USA, Cat. No 15140–122), in a humidified incubator at 37 °C with 5% CO_2_.

### 2.2. 3T3-L1 pre-adipocyte differentiation

**Fig. 1.**
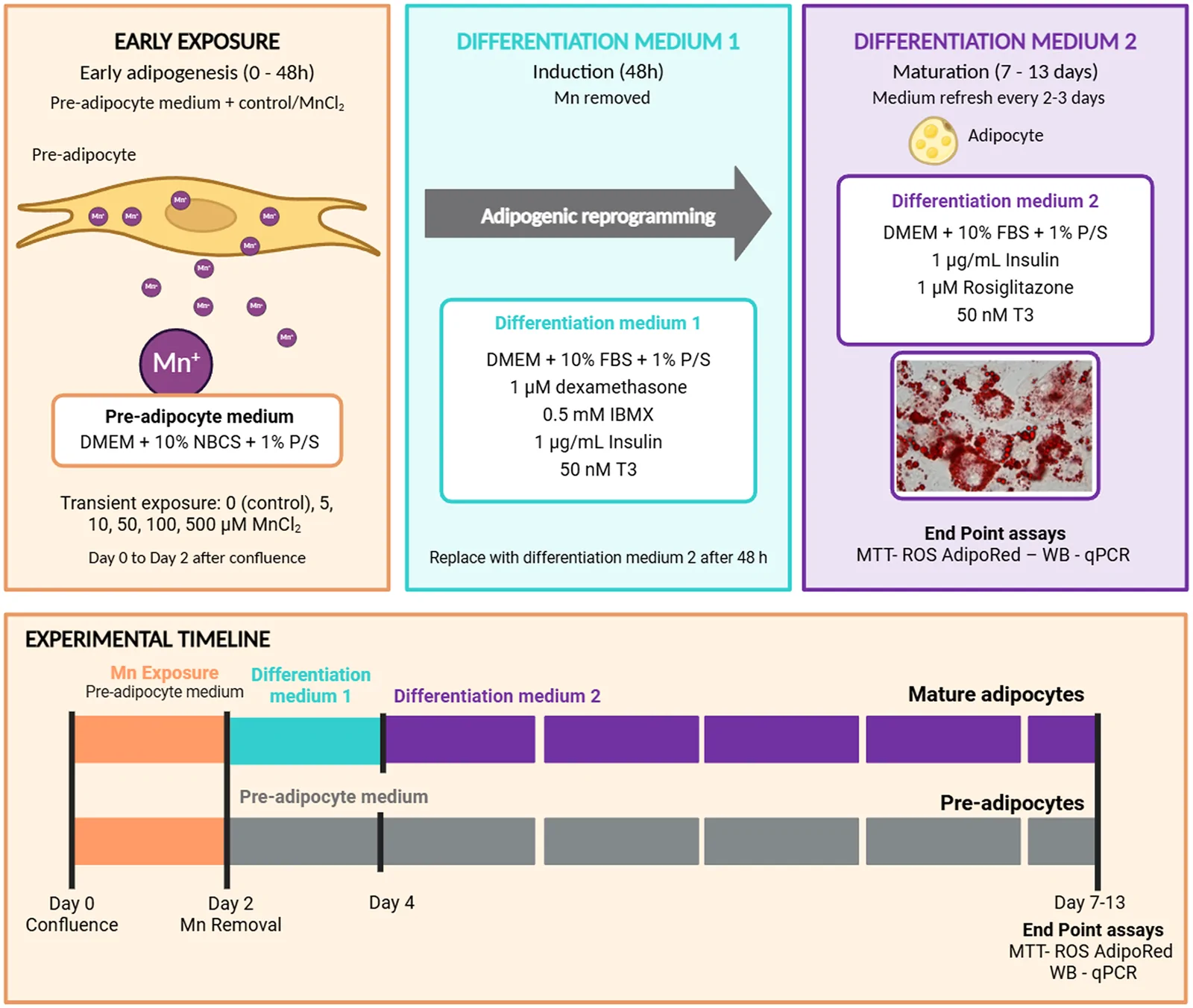
Manganese treatment and differentiation protocol. Upon reaching confluence, 3T3-L1 pre-adipocytes were exposed to different concentrations of Mn in pre-adipocyte medium for 48 h. Mn was then removed, and the medium was replaced with differentiation medium 1. After an additional 48 h, the medium was switched with differentiation medium 2 and refreshed every 2 or 3 days until full differentiation was achieved. A representative image of differentiated adipocytes generated using the protocol described in this study and stained with Oil Red O is shown. Pre-adipocytes exposed to Mn that were not differentiated, were maintained in pre-adipocyte medium for the same duration as differentiated adipocytes, with medium renewal every 2 or 3 days. Abbreviations: DMEM, Dulbecco’s Modified Eagle Medium; NBCS, newborn calf serum; FBS, fetal bovine serum; P/S, penicillin-streptomycin; IBMX, 3-isobutyl-1-methylxanthine; T3, 3,3′,5-triiodo-l-thyronine sodium salt. NBCS was used for pre-adipocyte maintenance, whereas FBS was used in differentiation media. Figure was generated using BioRender.com.

Differentiation was initiated 2 days after confluency was reached. Then the medium was replaced with differentiation medium 1, containing DMEM supplemented with 10% heat-inactivated fetal bovine serum (FBS, Gibco, USA, Cat. No A52568–01), 1% penicillin-streptomycin (Gibco, USA, Cat No.15140–122), 1 μM Dexamethasone (GBiosciences, USA, Cat No API-04), 0.5 mM 3-Isobutyl-1-methylxanthine (IBMX, Sigma-Aldrich, USA, Cat No I5879), 1 μg/mL insulin (Sigma-Aldrich, USA, Cat No I0516), and 50 nM 3,3′,5-Triiodo-l-Thyronine sodium salt (T3, Sigma-Aldrich, USA, Cat No T6397). 48 h later, the medium was replaced with differentiation medium 2, containing DMEM supplemented with 10% heat-inactivated fetal bovine serum (FBS, Gibco, USA, Cat. No A52568–01), 1% penicillin-streptomycin (Gibco, USA, Cat No 15140–122), 1 μg/mL insulin (Sigma-Aldrich, USA, Cat No I0516), 50 nM 3,3′,5-Triiodo-l-Thyronine sodium salt (T3, Sigma-Aldrich, USA, Cat No T6397), and 1 μM Rosiglitazone (Sigma-Aldrich, USA, Cat No R2408). Differentiation media were supplemented with 10% heat-inactivated FBS, which was the standardized serum preparation available and used consistently throughout the study. Medium was refreshed every 2 to 3 days. Cells were cultured in differentiation medium 2 until lipid droplet accumulation was evident (Fig. 1), with analyses performed between days 7 and 13 after induction depending on the independent differentiation experiment. Within each experiment, all Mn treatment groups and controls were analyzed in parallel on the same differentiation day. This protocol is a well-stablished differentiation protocol designed to generate 3T3-L1 adipocytes with white adipocyte morphology while promoting a brown/beige-like thermogenic phenotype. 3T3-L1 adipocytes differentiated in the presence of triiodothyronine (T3) and rosiglitazone have been reported to display a predominantly white phenotype with beige-like metabolic characteristics. (Asano, Kanamori et al., 2014; Bae and Kim, 2019).

### 2.3. Manganese treatment

Cells were subcultured into the appropriate plates for the different experiments. When cells reached confluency, pre-adipocytes were exposed to Manganese (II) Chloride (MnCl_2_, Sigma-Aldrich, USA, Cat. No 63535) at concentrations 0, 5, 10, 50, 100, and 500 μM for 48 h in pre-adipocyte medium. The initial MTT, ROS, and AdipoRed assays were performed using the full concentration range. Based on these results, 0, 5, 50, and 500 μM Mn were selected for subsequent molecular and functional analyses to include low, intermediate, and high Mn. After this transient exposure, manganese (Mn)-containing medium was removed, and cells were assigned to one of two experimental conditions. Cells in the non-differentiated condition were maintained in pre-adipocyte medium without adipogenic induction, whereas cells in differentiated condition were switched to differentiation medium 1 to generate mature adipocytes (Fig. 1). Both conditions included non-Mn-exposed controls and Mn-exposed groups. Thus, Mn exposure was limited to the period before hormonal induction and was not present during the differentiation or maturation phases.

The Mn concentrations were selected to capture the transition from physiologically essential levels to toxicological impairment. The lower concentration (5 μM) was chosen to mimic the recommendations of international regulatory agencies (Azab, Saleh et al., 2014; Martins, Krum et al., 2020; Efsa Panel on Nutrition, Food et al., 2023) and aligns with *in vitro* and *in vivo* studies regarding the beneficial effects of Mn supplementation (Mooney and Green, 1989; Burlet and Jain, 2013. In contrast the high concentration (500 μM) was chosen to simulate supraphysiological accumulation consistent with environmental or occupational exposures {Markiv, 2023 #85, Lee, Jouihan et al., 2013; Burlet and Jain, 2017; Dueck, Rafiee et al., 2021).

### 2.4. MTT assay

Mitochondrial reductase activity was assessed using the 3-(4,5-dimethylthiazol-2-yl)-2,5-diphenyltetrazolium bromide (MTT, Invitrogen, USA, Cat. No M6494) assay. Cells were seeded on a 96-well plate. When cells reached confluency, they were exposed to 0, 5, 10, 50, 100, and 500 μM Mn for 48 h. Then, Mn was removed and cells were maintained in pre-adipocyte medium or differentiated into adipocytes. Once clear lipid droplet accumulation was observed, the medium was replaced, and cells were incubated with 0.5 μg/μL MTT diluted in Hank’s Balanced Salt Solution (HBSS, Gibco, USA, Cat. No 14025–092) for 2 h in a humidified incubator at 37 °C with 5% CO_2_. After 2 h, the MTT solution was removed, and the formazan crystals produced by the reduction of MTT by metabolically active cells were dissolved using dimethyl sulfoxide (DMSO, Sigma-Aldrich, USA, Cat. No D8418). Absorbance was measured at 540 nm using a SpectraMax iD3 (Molecular Devices) plate reader. No background correction or blank subtraction was applied. Control wells were used only to verify assay performance, and MTT data were analyzed using raw absorbance values at 540 nm.

### 2.5. Reactive oxygen species levels measurement

Reactive oxygen species (ROS) production was measured using the cell-permeable 2′,7′-dichlorodihydrofluorescein diacetate (CM-H2DCFDA) probe (Invitrogen, USA, Cat. No C6827). Cells were seeded on a black 96-well plate. When cells reached confluency, they were exposed to 0, 5, 10, 50, 100, and 500 μM Mn for 48 h. Then, Mn was removed and cells were maintained in pre-adipocyte medium or differentiated into adipocytes. Once clear lipid droplet accumulation was observed, cells were washed with HBSS and incubated for 30 min with 10 μM CM-H_2_DCFDA in a humidified incubator at 37 °C with 5% CO_2_. CM-H_2_DCFDA stock solutions were prepared in DMSO and diluted immediately before use. Fluorescence intensity was measured at 495 nm excitation and emission of 535 nm with a SpectraMax iD3 (Molecular Devices) plate reader.

### 2.6. Lipid accumulation assay

Lipid accumulation in adipocytes was measured using the AdipoRed^™^ assay reagent (Lonza, USA, Cat. No PT-7009) following manufacturer’s instructions. Briefly, cells were seeded into a white 96-well plate. When cells reached confluency, they were exposed to 0, 5, 10, 50, 100, and 500 μM Mn for 48 h. Then, Mn was removed and cells were maintained in pre-adipocyte medium or differentiated into adipocytes. Once clear lipid droplet accumulation was observed, the medium was removed, and cells were rinsed with phosphate buffered saline (PBS, Gibco, USA, Cat. No 10010–031). Cells were then incubated for 10 min at room temperature with 5 μL of AdipoRed^™^ reagent added to 200 μL PBS per well. Finally, fluorescence was measured with excitation at 485 nm and emission at 572 nm using a SpectraMax iD3 (Molecular Devices) plate reader.

### 2.7. Western blot analysis

Cells were seeded in 60 mm plates and treated and differentiated. Once clear lipid droplet accumulation was observed, cells were washed using Phosphate Buffered Saline (PBS, Gibco, USA, Cat. No 10010–031) Cells were lysed using cold radioimmunoprecipitation (RIPA, Thermo Fisher Scientific, USA, Cat. No 89901) buffer supplemented with phosphatase inhibitor cocktail 2 (Sigma-Aldrich, Cat. USA, No P5726), phosphatase inhibitor cocktail 3 (Sigma-Aldrich, USA, Cat. No P0044), and Halt^™^ protease inhibitor cocktail (Thermo Fisher Scientific, USA, Cat. No 78437). Cell lysates were sonicated using a Sonics ultrasonic processor (Sonics, VCX130–220 V) and centrifuged at 10,000 xg for 10 min at 4 °C. Protein concentration was measured with the Pierce^™^ BCA Protein Assay Kit (Thermo Fisher Scientific, USA, Cat. No 23227).

Samples were diluted with Laemmli buffer (Bio-Rad, USA, Cat. No 1610737) and heated for 5 min at 100 °C. Protein (20 μg) was loaded onto a Mini-PROTEAN TGX Gel (Bio-Rad, USA, Cat. No 4561096 or 4561024) and separated using Mini-PROTEAN Tetra Vertical Electrophoresis Cell (Bio-Rad, USA, Cat. No 1658005) by Sodium Dodecyl Sulfate–Polyacrylamide Gel Electrophoresis (SDS-PAGE). Proteins were transferred onto a nitrocellulose membrane (Amersham^™^ Protran^™^ 0.2 μM NC, USA, Cat. No 10600006). Membranes were blocked for 1 hour at room temperature with 5% bovine serum albumin (BSA, Fisher Scientific, USA, Cat. No BP1600–100) diluted in Tris buffer saline (TBS, Bio-Rad, Cat. No.1706435) containing 0.1% Tween-20 (Promega, Cat. No H5151) (TBS-T) to block non-specific binding sites. Subsequently, membranes were incubated overnight at 4 °C with primary antibody. Membranes were then washed with TBS-T three times and incubated with appropriate secondary antibodies for 1 hour. Protein bands were developed using SuperSignal^™^ WestPico Plus Chemiluminescent Substrate (Thermo Fisher Scientific, Cat. No 34580) and visualized using the ChemiDoc^™^ Touch Gel Imaging System (Bio-Rad). Protein bands were quantified using ImageJ software (NIH). The following antibodies were used: ACC (Cell Signaling Technology, USA, Cat# 3676, RRID: AB_2219397), phospho-ACC (Cell Signaling Technology, USA, Cat# 3661, RRID:AB_330337), Adiponectin (Cell Signaling Technology, USA, Cat# 2789, RRID:AB_2221630), AMPKα (Cell Signaling Technology, USA, Cat# 2532, RRID:AB_330331), phospho-AMPKα (Cell Signaling Technology,USA, Cat# 2535, RRID:AB_331250), AKT (Cell Signaling Technology, USA, Cat# 4691, RRID:AB_915783), C/EBPα (Cell Signaling Technology, USA, Cat# 8178, RRID:AB_11178517), FAS (Cell Signaling Technology, USA, Cat# 3180, RRID:AB_2100796), HSL (Cell Signaling Technology, USA, Cat# 18381, RRID:AB_2798800), phospho-HSL (Ser563) (Cell Signaling Technology, USA, Cat# 4139, RRID: AB_2135495), phospho-HSL (Ser565) (Cell Signaling Technology, USA, Cat# 4137, RRID:AB_2135498), phospho-HSL (Ser660) (Cell Signaling Technology, USA Cat# 45804, RRID:AB_2893315), perilipin-1 (Cell Signaling Technology, USA, Cat# 9349, RRID:AB_10829911), and PPARγ (Cell Signaling Technology, USA, Cat# 2435, RRID: AB_2166051). To normalize protein expression levels, β-actin (Sigma-Aldrich Cat# A1978, RRID:AB_476692) was used. Because β-actin expression may vary during adipogenesis, comparisons of normalized protein levels across differentiation states should be interpreted with caution.

### 2.8. RNA extraction and real-time quantitative PCR

During the confluency phase, cells were exposed to 0, 5, 50, or 500 μM Mn for 48 h. Following this exposure, the Mn-containing medium was removed, and cells were maintained in pre-adipocyte medium or differentiated into adipocytes. Total RNA was isolated using the Qiagen RNeasy Mini kit (Qiagen, Germany, Cat .No. 74104) following manufacturer’s instructions. RNA concentration and purity were assessed using a Nanodrop 2000 Spectrophotometer (Thermo Fisher Scientific, USA, Cat. No ND2000) based on A260/A280 absorbance ratios before cDNA synthesis. Complementary DNA (cDNA) was synthesized using the Applied Biosystems High-Capacity cDNA Reverse Transcription Kit (Applied Biosystems, USA, Cat. No 4368814) according to the manufacturer’s instructions. Quantitative PCR (qPCR) was performed on a CFX96 Real-Time System Thermal Cycler using BioRad CFX manager software. Gene expression was normalized to glyceraldehyde 3-phosphate dehydrogenase (*Gapdh*) and calculated using the 2^**−ΔΔ**Ct^ method (Livak and Schmittgen, 2001). Gapdh was used as the sole internal reference gene; therefore, RT-qPCR comparisons across differentiation states should be interpreted with caution because Gapdh stability was not formally validated across all experimental groups.

The following commercially available, manufacturer-validated Taq-Man primers from Applied Biosystems were used: *Acaca* (Cat. No Mm01304257_m1), *Cpt1a* (Cat. No Mm01231183_m1), *Fasn* (Cat. No Mm00662319_m1), *Hmox1* (Cat. No Mm00516005_m1), *Insr* (Cat. No Mm01211875_m1), *Lep* (Cat. No Mm00440181_m1), *Lepr* (Cat. No Mm00434759_m1), *Lpl* (Cat. No Mm00434764_m1), *Nqo1* (Cat. No Mm01253561_m1), *Pnpla2* (Cat. No Mm00503040_m1), *Pparg* (Cat. No Mm00440945_m1), *Prdm16* (Cat. No Mm00712556_m1), *Slc2a1* (Cat. No Mm00441480_m1), *Slc2a4* (Cat. No Mm00436615_m1), *Slc7a11* (Cat. No Mm00442530_m1), *Sod2* (Cat. No Mm01313000_m1), and *Ucp1* (Cat. No Mm01244861_m1).

### 2.9. Lipolysis stimulation cell sample collection

To determine the persistent effects of Mn on lipolysis, cells were washed twice with PBS and incubated with 0.5 mL Krebs-Ringer HEPES buffered solution (KRHB, Thermo Fisher Scientific, USA, J67795.AP) supplemented with 2% fatty acid free bovine serum albumin (Sigma-Aldrich, USA, A1595) for 3 h at 37 °C in a humidified incubator with 5% CO_2_. Lipolysis was then induced using 10 μM of the non-selective β-adrenergic receptor agonist isoproterenol (ISO; Cayman Chemical, USA, Cat. No 15592) or 10 μM of the selective β_3_- adrenergic receptor agonist CL 316,243 (CL; Cayman Chemical, USA, Cat. No 17499) for 30 min. Conditioned media were collected to measure glycerol release, and cells were lysed in RIPA buffer for protein expression analysis by western blot.

### 2.10. Glycerol measurements

The release of glycerol in the medium was measured using the EnzyChrom^™^ Adipolysis Assay Kit (BioAssay Systems, USA, Cat. No EAPL-200) according to the manufacturer’s protocol. Briefly, 10 μL of culture medium and 100 μL of enzyme reaction mixture, consisting of 2 μL Enzyme Mix, 1 μL ATP, and 1 μL Dye Reagent, were transferred to a 96-well plate and incubated at room temperature for 20 min. Finally, absorbance was read at 570 nm with a SpectraMax iD3 (Molecular Devices) plate reader. To account for variations in cell density, glycerol release was normalized to total protein content evaluated using Pierce^™^ BCA Protein Assay Kit (Thermo Fisher Scientific, USA, Cat. No 23227).

### 2.11. Statistical analysis

Statistical analyses were performed using IBM Statistical Package for the Social Sciences (SPSS) version 30.0.00 (172) software. GraphPad Prism version 10.6.1 (892) software was used for graphical data representation. No sample size calculations were carried out. No data point was excluded, as no test for outliers was performed. The Shapiro-Wilk test and the Kolmogorov-Smirnov test were used to test for data normality.

The effects of Mn in non-differentiated (pre-adipocytes) and differentiated (adipocytes) cells on MTT metabolic activity, oxidative stress, lipid accumulation, glycerol release, protein and gene expression were assessed by two-way analysis of variance (ANOVA), followed by Bonferroni’s *post hoc* test. F values were indicated as F_Mn_ (Manganese significant effect), F_dif_ (Differentiation significant effect), and F_Int_ (Manganese × differentiation interaction significant effect).

Data from lipolysis experiments were also analyzed using a two-way ANOVA, followed by Bonferroni’s *post hoc* test. F values were indicated as F_Mn_ (Manganese significant effect), F_ago_ (β-adrenergic receptor agonist significant effect), and F_Int_ (Manganese × agonist interaction significant effect)

For datasets that did not meet normality assumptions, logarithmic or square-root transformation were applied to improve distributional normality. Data are reported as mean ± standard deviation (SD), and statistical significance was set at p < 0.05. For each assay, individual data points represent independent biological replicates from separate cell cultures processed in parallel.

## 3. Results

### 3.1. Early manganese exposure persistently reduces MTT metabolic activity without sustained effects on ROS production

**Fig. 2.**
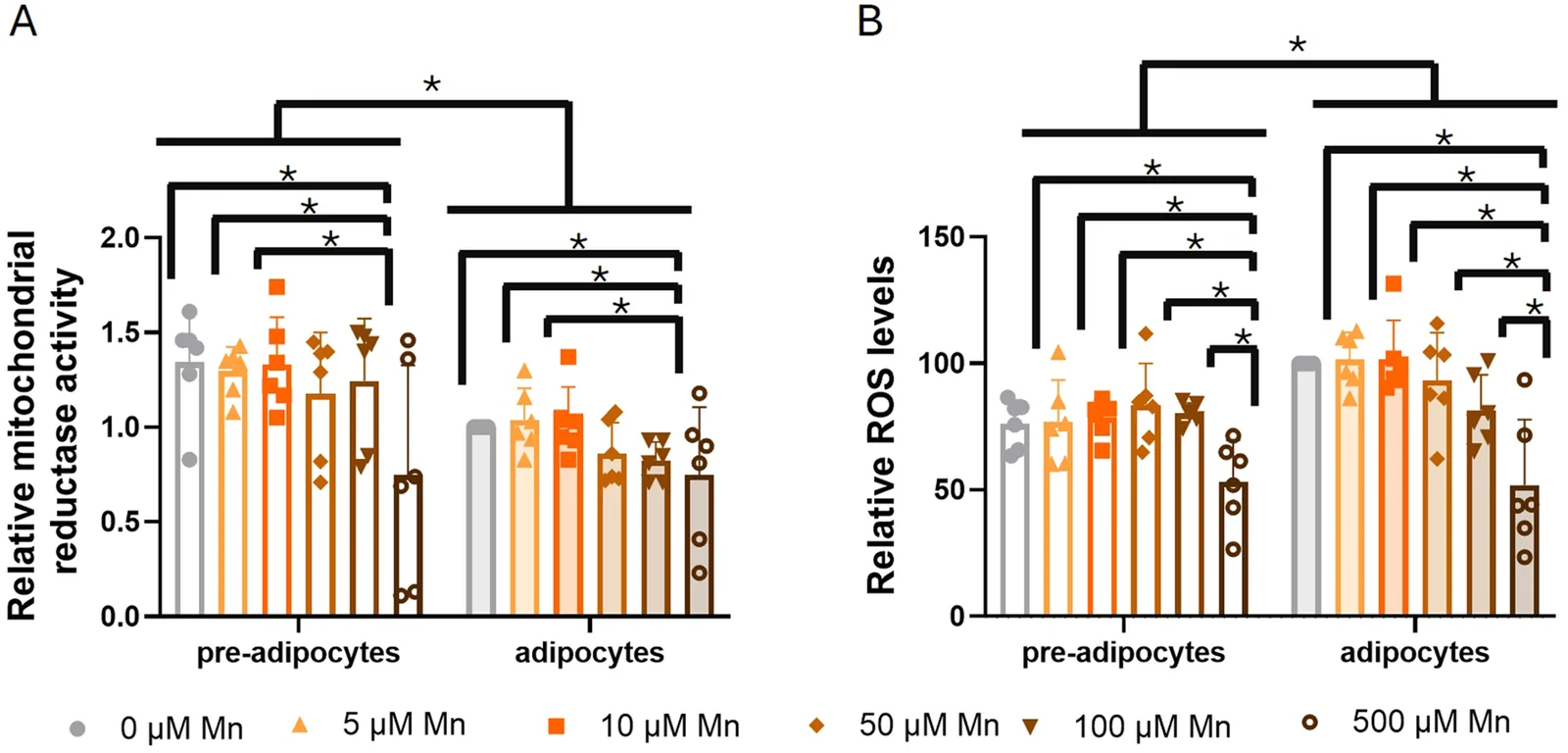
Early Mn exposure attenuates mitochondrial reductase activity despite the absence of sustained oxidative stress. **(A)** Mitochondrial reductase activity (MTT assay) and **(B)** ROS production in pre-adipocyte and fully differentiated adipocytes transiently exposed to Mn (0, 5, 10, 50, 100, or 500 μM) during the confluence phase. Data are presented as mean ± SD. Statistical significance was determined using two-way ANOVA followed by Bonferroni’s post hoc test. *P* < 0.05 was considered statistically significant. * indicates significant differences. Each point represents an independent biological replicate from a separate cell culture.

To study the long-term impact of transient Mn exposure on adipocyte cells, we investigated mitochondrial metabolic activity in both non-differentiated (pre-adipocytes) and differentiated (adipocytes) 3T3-L1 cells. Cells were exposed to Mn for 48 h during the confluency phase, after which Mn was removed, and the differentiation protocol was initiated. Mitochondrial metabolic activity, assessed via the MTT assay, was significantly lower in fully differentiated adipocytes compared to pre-adipocytes (F_dif_(1, 60) = 17.405, p < 0.001) (Fig. 2A). This likely reflects the physiological transition from a highly proliferative metabolic state in pre-adipocytes to a quiescent, storage-focused state in mature adipocytes. Notably, the highest Mn concentrations induced a persistent impairment of mitochondrial reductase activity that remained evident after Mn removal (F_Mn_(5, 60) = 4.262, p = 0.002) (Fig. 2A). Because MTT reflects cellular reductive/metabolic activity but was not normalized to cell number, DNA content, or total protein content, reductions observed at the highest Mn concentration may reflect decreased metabolic activity, reduced viable cell number, or both. Therefore, downstream effects at 500 μM Mn should be interpreted with caution, as they may indicate persistent metabolic impairment and possible toxicity.

Conversely, two-way ANOVA identified an overall effect of differentiation on ROS levels, with differentiated adipocytes showing higher ROS signals than pre-adipocytes (F_dif_(1, 60) = 15.613, p < 0.001) (Fig. 2B). This difference was primarily evident in the unexposed and lower Mn concentration groups and was no longer apparent at 100 and 500 μM Mn. Mn concentrations from 5 to 50 μM did not significantly alter ROS levels relative to the corresponding unexposed condition. In contrast, higher Mn concentrations were associated with a persistent reduction in ROS signal (F_Mn_(5, 60) = 12.106, p < 0.001). Because high-Mn concentrations also reduced MTT metabolic activity, the lower ROS signal at high concentrations may reflect reduced metabolic activity or fewer metabolically active cells rather than lower oxidative burden per cell.

### 3.2. Early manganese exposure induces persistent antioxidant stress-response changes in differentiated adipocyte

**Fig. 3.**
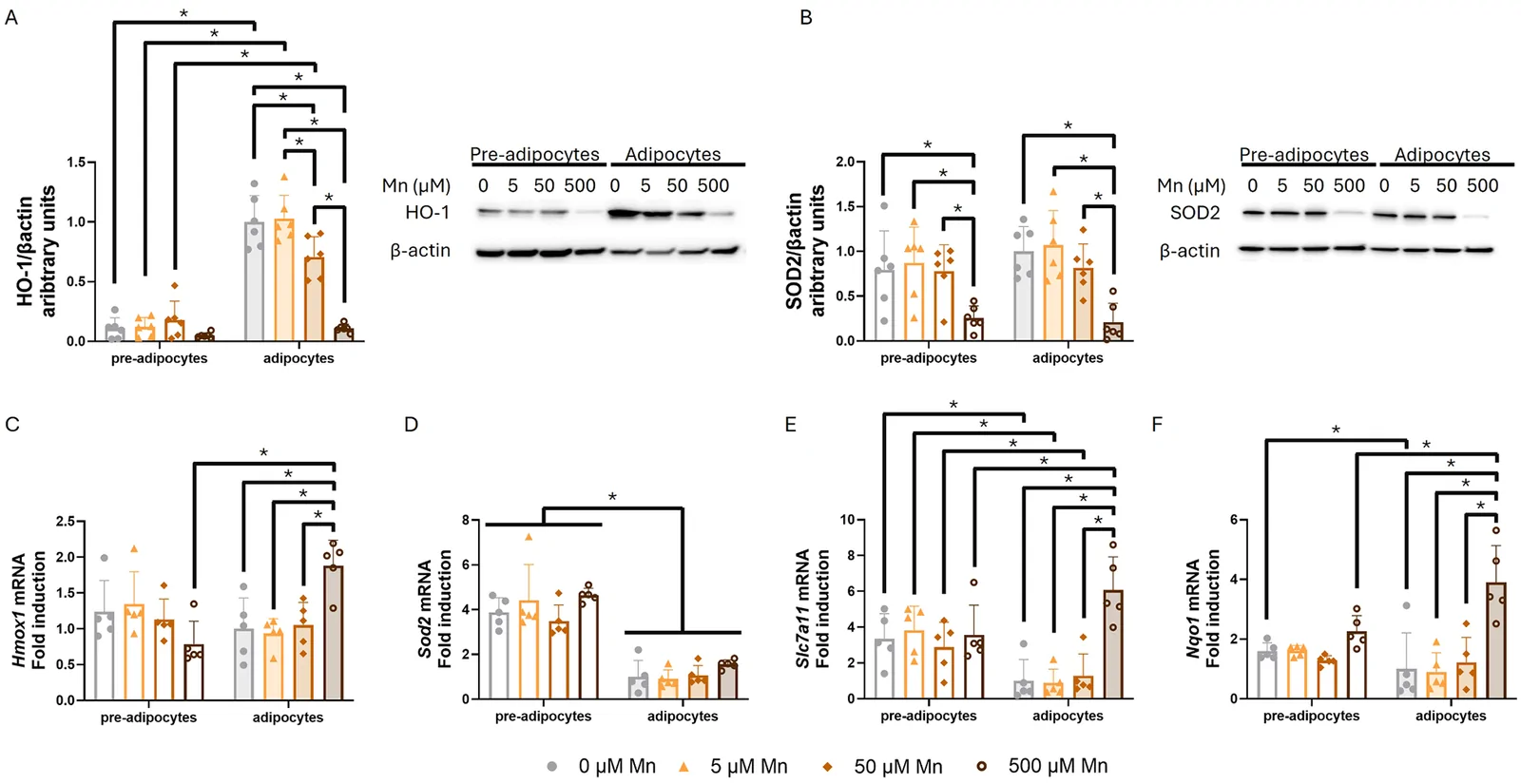
Early Mn exposure persistently impairs antioxidant defense systems. Pre-adipocytes were transiently exposed to Mn (0, 5, 50, or 500 μM) during the confluence phase. Following Mn removal, cells were either maintained in pre-adipocyte medium or differentiated into mature adipocytes. Protein levels and gene expression of antioxidant enzymes were quantified in pre-adipocytes and in fully differentiated adipocytes using western blot and qPCR analysis, respectively. **(A)** HO-1 protein levels quantified by densitometry and representative western blot. **(B)** SOD2 protein levels quantified by densitometry and representative western blot. **(C)** *Hmox1*, **(D)** *Sod2*, **(E)** *Slc7a11*, and **(F)** *Nqo1* mRNA expression levels. Data are presented as mean ± SD. Statistical significance was determined using two-way ANOVA followed by Bonferroni’s post hoc test. *P* < 0.05 was considered statistically significant. * indicates significant differences. Each point represents an independent biological replicate from a separate cell culture.

To investigate the mechanisms underlying the observed changes in oxidative stress, we analyzed the expression of key antioxidant enzymes. Fully differentiated adipocytes exhibited significantly higher hemeoxygenase-1 (HO-1) protein levels than pre-adipocytes, consistent with differentiation-associated differences in redox regulation. However, early Mn exposure resulted in a persistent, concentration-dependent decrease in HO-1 protein levels exclusively in differentiated adipocytes (F_int_(3, 40) = 24.580, p < 0.001) (Fig. 3A).

Mn is an essential catalytic cofactor of manganese superoxide dismutase (MnSOD or SOD2), a primary mitochondrial enzyme responsible for dismutation of the superoxide radical. Notably, adipocyte differentiation had no effect on SOD2 protein levels, while early Mn exposure resulted in a significant, persistent decrease in its expression (F_Mn_(3, 40) = 13.518, p < 0.001) (Fig. 3B). These findings demonstrate that early Mn exposure consistently reduces SOD2 protein level across both adipocyte stages, reflecting a persistent impairment of basal antioxidant defenses, whereas the reduction of HO-1 protein occurs only in differentiated adipocytes, suggesting that early Mn exposure impairs antioxidant defenses in adipocytes in a stage-dependent manner.

In contrast to the protein data, *Hmox1* gene expression was not affected by differentiation. However, fully differentiated adipocytes previously exposed to the highest dose of Mn presented a significant increase in *Hmox1* mRNA expression compared to pre-adipocytes under the same conditions (F_int_(3, 32) = 8.540, p < 0.001, *post hoc* p < 0.001) (Fig. 3C). While early Mn exposure did not affect *Hmox1* gene expression in pre-adipocytes, it induced an upregulation in fully differentiated adipocytes that reached statistical significance only at 500 μM Mn (*post hoc*p = 0.008 compared with differentiated adipocytes without Mn exposure) (Fig. 3C).

Notably, *Sod2* gene expression did not mirror the protein changes. While *Sod2* gene expression was significantly lower in mature adipocytes compared to pre-adipocytes (F_dif_(1, 32) = 193.638, p < 0.001) (Fig. 3D), early Mn exposure had no long-term effects on *Sod2* mRNA expression. This divergence suggests that Mn-induced reduction in SOD2 is not mediated by suppression of gene transcription.

Similar long-lasting effects were observed for other antioxidant genes. Early exposure to 500 μM Mn significantly upregulated solute carrier family 7 member 11 (*Slc7a11*) ((F_int_(3, 32) = 7.778, p < 0.001) and NAD(P)H quinone dehydrogenase 1 (*Nqo1*) (F_int_(3, 32) = 4.608, p = 0.009) mRNA levels in fully differentiated adipocytes (*post hoc*p < 0.001 compared with differentiated adipocytes without Mn exposure for both genes), whereas no statistically significant differences were observed at 5 or 50 μM Mn (Fig. 3E and F). Pre-adipocytes remained unaffected. Interestingly, differentiated adipocytes exposed to the highest concentration of Mn showed higher *Slc7a11* gene expression than pre-adipocytes exposed to the same dose. In contrast, at lower Mn concentrations, *Slc7a11* mRNA expression was reduced in mature adipocytes compared with pre-adipocytes (*post hoc* p = 0.002) (Fig. 3E). Similarly, mature adipocytes not exposed to Mn showed significantly lower *Nqo1* gene expression than non-exposed pre-adipocytes (*post hoc*p = 0.049), whereas early exposure to 500 μM Mn concentration resulted in a significant increase in *Nqo1* gene expression in mature adipocytes (*post hoc*p = 0.017 compared with pre-adipocytes exposed to 500 μM Mn concentration) (Fig. 3F).

Together, these results indicate concentration- and differentiation-dependent changes in antioxidant markers, with the significant transcriptional effects in mature adipocytes observed only after exposure to 500 μM Mn.

### 3.3. Early manganese exposure impairs lipid accumulation and basal lipolysis in fully differentiated adipocytes

**Fig. 4.**
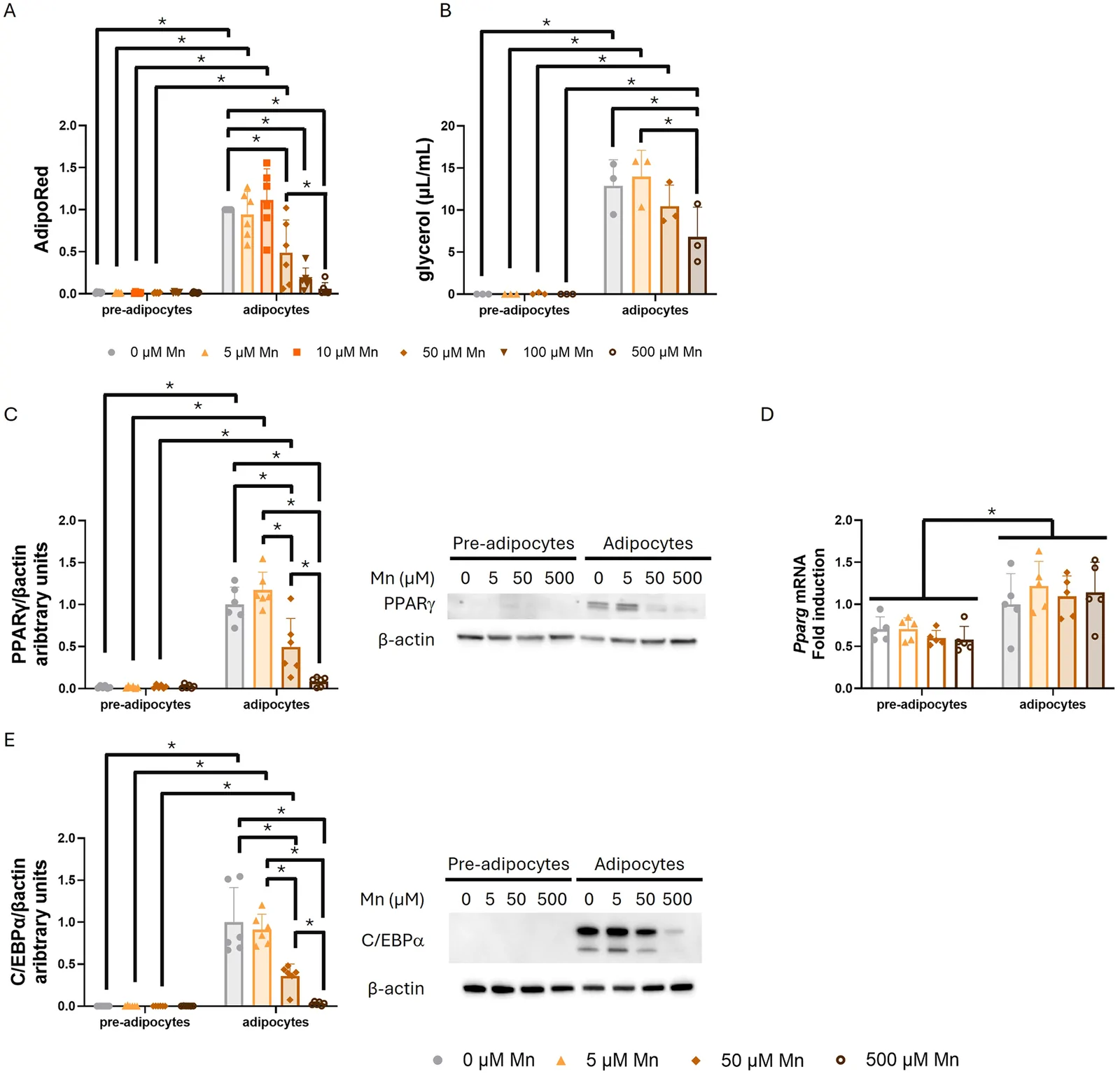
Transient Mn exposure during the confluence phase leads to persistent deficits in lipid storage and basal lipolysis and impairs adipogenic transcription factor expression. Pre-adipocytes were transiently exposed to Mn during the confluence phase. After Mn removal, cells were either maintained in pre-adipocyte medium or differentiated into mature adipocytes. **(A)** Lipid accumulation was quantified using AdipoRed^™^ staining in cells transiently exposed to Mn (0, 5, 10, 50, 100, or 500 μM). **(B)** Basal lipolysis was assessed by measuring unstimulated glycerol release into the culture medium in pre-adipocytes and fully differentiated adipocytes transiently exposed to Mn (0, 5, 50, or 500 μM). Protein and mRNA expression of key adipogenic transcription factors were analyzed by western blot and qPCR, respectively, in cells transiently exposed to Mn (0, 5, 50, or 500 μM). **(C)** PPARγ protein levels quantified by densitometry and representative western blot. **(D)** *Pparg* mRNA expression. **(E)** C/EBPα protein levels quantified by densitometry and representative western blot. Data are presented as mean ± SD. Statistical significance was determined using two-way ANOVA followed by Bonferroni’s post hoc test. *P* < 0.05 was considered statistically significant. * indicates significant differences. Each point represents an independent biological replicate from a separate cell culture.

During the differentiation of 3T3-L1 pre-adipocytes into an adipocyte phenotype, the cells undergo a dramatic morphological transformation, transitioning from an elongated, fibroblastic shape to a spherical, enlarged phenotype. The hallmark of this maturation is the development of large intracellular lipid droplets containing triglycerides. Representative Oil Red O lipid staining is included in Fig. 1, showing lipid droplet accumulation under the differentiation conditions used in this study. To further confirm successful differentiation and evaluate the long-term impact of Mn exposure, lipid content was quantified using AdipoRed^™^ staining. As expected, differentiated adipocytes presented significantly higher lipid content than pre-adipocytes, which was evidenced by an increase in AdipoRed^™^ fluorescence. However, early Mn exposure resulted in a persistent, concentration-dependent decrease in lipid accumulation, with significant reductions observed at 50, 100, and 500 μM Mn (F_int_(5, 60) = 18.964, p < 0.001) (Fig. 4A).

Under basal (resting) conditions, glycerol levels in the culture medium, an indicator for the continuous triglyceride hydrolysis, were substantially higher in differentiated adipocytes than in pre-adipocytes. This elevated basal lipolysis correlates with the high cellular lipid content characteristic of mature adipocytes. Notably, early Mn exposure significantly reduced basal glycerol levels in fully differentiated adipocytes. This reduction was significant at 500 μM Mn (F_int_(3, 16) = 3.245, p = 0.05, *post hoc*p = 0.019 compared with non-exposed differentiated adipocytes) (Fig. 4B).

Taken together, these results demonstrate that Mn exposure restricted to the confluency phase causes persistent concentration-related deficits in both the accumulation and mobilization of lipids in mature adipocytes. These data support a persistent effect of early Mn exposure on adipocyte maturation at lower and intermediate concentrations, whereas the highest concentration likely includes a substantial stress or toxicity.

### 3.4. Early manganese exposure during differentiation impairs adipogenic transcription factors in fully differentiated adipocytes

The reduction in lipid content following early Mn exposure could result from disrupted lipid metabolisms or impaired adipogenesis. The differentiation of pre-adipocytes into mature adipocytes is a tightly regulated process; thus, the long-lasting effects of Mn on lipid accumulation may reflect alterations in key adipogenic pathways.

The protein levels of peroxisome proliferator-activated receptor gamma (PPARγ), the master regulator of adipogenesis, were significantly higher in fully differentiated adipocytes compared to pre-adipocytes (F_int_(3, 40) = 28.727, p < 0.001) (Fig. 4C). However, early Mn exposure selectively decreases PPARγ protein expression in differentiated adipocytes, with significant decreases observed at 50 and 500 μM Mn (Fig. 4C). In contrast, while *PPAR gamma* (*Pparg*) mRNA expression increased with differentiation (F_dif_(1, 32) = 36.057, p < 0.001), no Mn-induced effects were detected at transcriptional level (Fig. 4D), suggesting a post-transcriptional impact of Mn on this transcription factor.

PPARγ activates CCAAT/enhancer-binding protein alpha (C/EBPα), forming a positive feedback loop essential for complete adipocyte maturation. Consistent with PPARγ data, C/EBPα protein expression increased upon differentiation, but was persistently suppressed by early Mn exposure in a concentration dependent-manner (F_int_(3, 40) = 22.637, p < 0.001). Significant reductions were observed starting at 50 μM Mn (post hoc p < 0.001 compared with non-treated differentiated adipocytes) (Fig. 4E). At the highest concentration (500 μM), C/EBPα protein levels were further reduced. These results suggest that early Mn exposure may affect the adipogenic program in a concentration-related manner, which could contribute to the reductions observed in lipid accumulation.

### 3.5. Early manganese exposure during differentiation alters adipokine expression in fully differentiated adipocytes

**Fig. 5.**
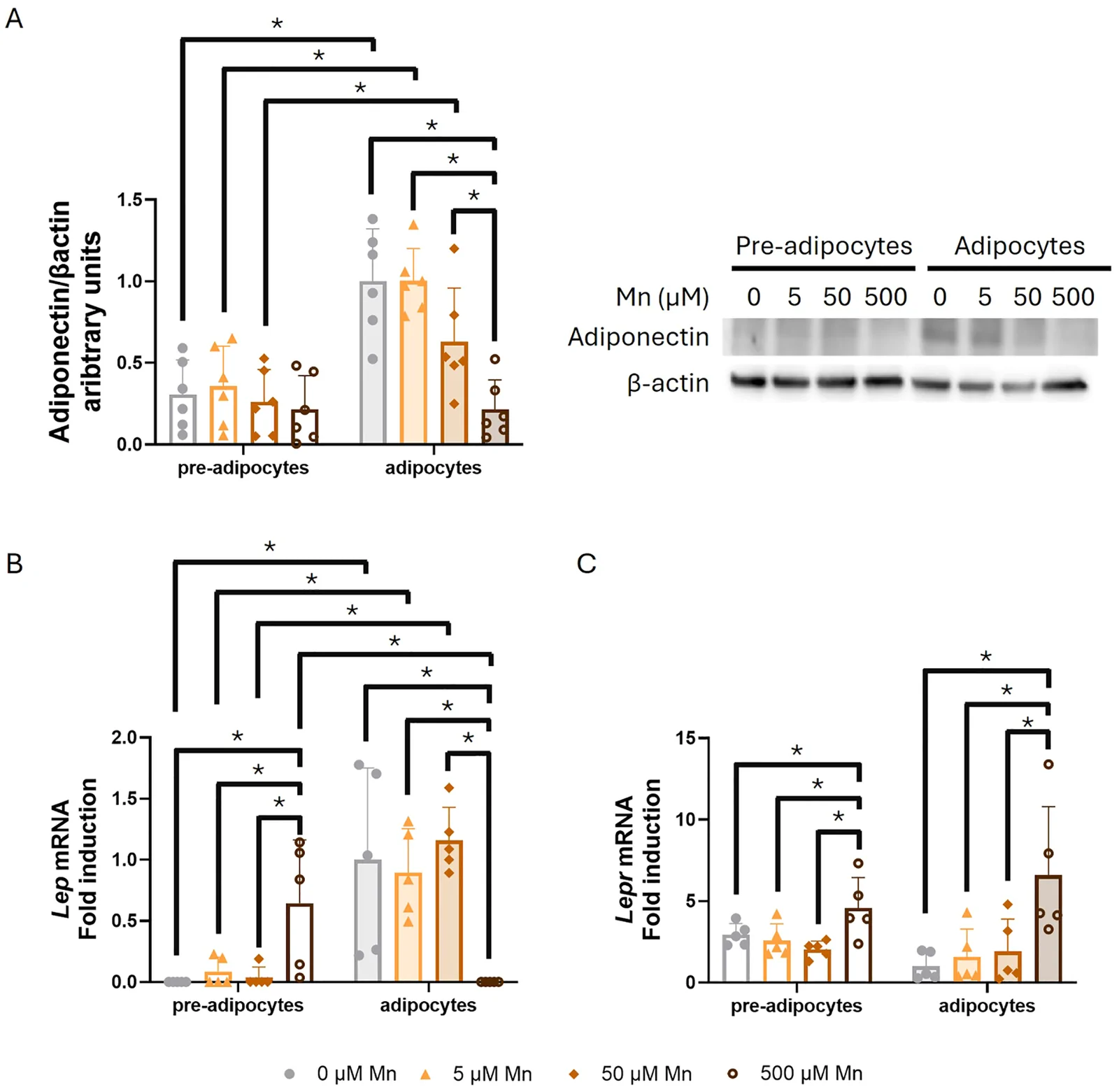
Transient Mn exposure disrupts adipokine expression in pre-adipocytes and fully differentiated adipocytes. Pre-adipocytes were transiently exposed to Mn (0, 5, 50, or 500 μM) during the confluence phase. After Mn removal, cells were either maintained in pre-adipocyte medium or differentiated into mature adipocytes. Protein and mRNA levels of key adipokines were analyzed by western blot and qPCR, respectively. **(A)** Adipokine protein levels quantified by densitometry and representative western blot. **(B)** *Leptin* mRNA expression. **(C)**. *Leptin receptor* mRNA expression. Data are presented as mean ± SD. Statistical significance was determined using two-way ANOVA followed by Bonferroni’s post hoc test. *P* < 0.05 was considered statistically significant. * indicates significant differences. Each point represents an independent biological replicate from a separate cell culture.

As 3T3-L1 cells progressed through differentiation, they began to produce and secrete adipokines, which regulate metabolic homeostasis and positively reinforce adipogenesis. Adipokines such as adiponectin and leptin are widely used as functional markers of mature adipocytes. Early Mn exposure caused a persistent reduction in adiponectin protein levels in fully differentiated adipocytes, with significant reductions observed at 500 μM, Mn despite these cells exhibiting higher adiponectin protein expression than pre-adipocytes (F_int_(3, 40) = 3.103, p = 0.037) (Fig. 5A).

Similarly, *leptin* (*Lep*) mRNA expression increased with differentiation, but early Mn exposure produced divergent, state-dependent effects (F_int_(3, 32) = 26.561, p < 0.001). Specifically, Mn increased *Lep* gene expression in pre-adipocytes but significantly decreased it in fully differentiated adipocytes (Fig. 5B). While the *leptin receptor* (*Lepr*) gene expression was unaffected by differentiation process (Fig. 5C), early Mn exposure significantly upregulated *Lepr* expression in both pre-adipocytes and mature adipocytes (F_Mn_(3, 32) = 8.991, p < 0.001) (Fig. 5C).

These data demonstrate that early Mn exposure induces long-lasting disruption of the adipokine gene expression, suggesting that Mn-exposed cells fail to reach full metabolic maturity.

### 3.6. Early manganese exposure during differentiation interferes with the transcriptional transition from GLUT1 to GLUT4 and alters insulin receptor gene expression

**Fig. 6.**
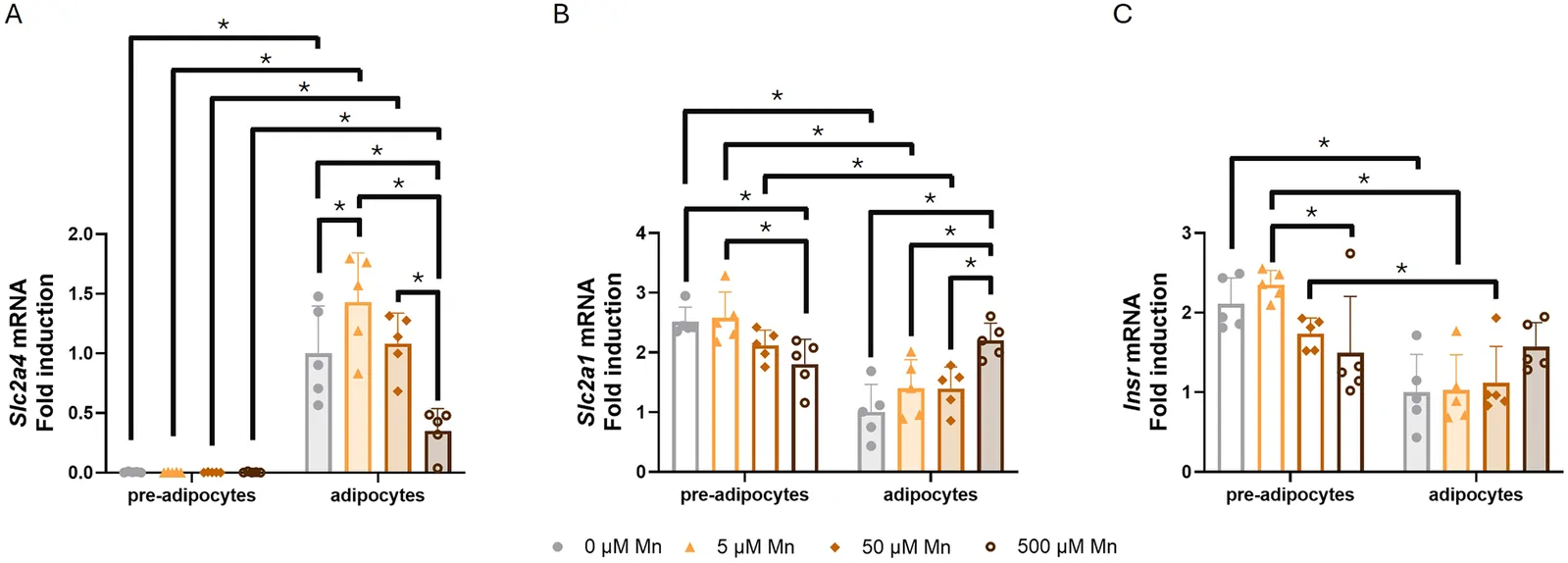
Early Mn exposure during differentiation disrupts the GLUT1-to-GLUT4 transcriptional transition and insulin receptor expression. Pre-adipocytes were transiently exposed to Mn (0, 5, 50, or 500 μM) during the confluence phase. After Mn removal, cells were either maintained in pre-adipocyte medium or differentiated into mature adipocytes. mRNA expression of glucose transporters and insulin receptor were analyzed by qPCR. **(A)** *Slc2a4* (GLUT4) mRNA expression. **(B)** *Slc2a1* (GLUT1) mRNA expression. **(C)** *Insulin receptor* mRNA expression. Data are presented as mean ± SD. Statistical significance was determined using two-way ANOVA followed by Bonferroni’s post hoc test. *P* < 0.05 was considered statistically significant. * indicates significant differences. Each point represents an independent biological replicate from a separate cell culture.

A hallmark of adipocyte maturation is the expression of glucose transporter 4 (GLUT4), encoded by the *solute carrier family 2 member 4* (*Slc2a4*) gene, which mediates insulin-stimulated glucose uptake. As expected, *Slc2a4* mRNA were negligible in pre-adipocytes but highly expressed in mature adipocytes. Early Mn exposure exerted divergent, dose-dependent effects on mature adipocytes: whereas the lowest concentration (5 μM) significantly increased *Slc2a4* expression, the highest concentration (500 μM) significantly decreased it (F_int_(3, 32) = 9.487, p < 0.001, *post hoc*p = 0.036 for 5 μM Mn compared with non-exposed differentiated adipocytes and p < 0.001 for 500 μM Mn compared with non-exposed differentiated adipocytes) (Fig. 6A).

In contrast, basal glucose transport is primarily mediated by glucose transporter 1 (GLUT1), encoded by *solute carrier family 2 member 1* (*Slc2a1)* gene. We observed that *Slc2a1* mRNA expression was highest in pre-adipocytes and declined during differentiation. Early Mn exposure disrupted this pattern. In pre-adipocytes, Mn decreased *Slc2a1* gene expression, whereas in mature adipocytes it produced the opposite effect, significantly increasing its expression at 500 μM Mn (F_int_(3, 32) = 12.084, p < 0.001) (Fig. 6B).

Insulin receptor (INSR)-mediated signaling is essential for the transition from GLUT1 to GLUT4 and the promotion of adipogenesis. Fully differentiated adipocytes exhibited lower *Insr* mRNA levels than pre-adipocytes. Early Mn exposure significantly reduces *Insr* gene expression in pre-adipocytes at 500 μM Mn, though it had no effect on mature cells. Interestingly, pre-adipocytes exposed to the highest concentration presented similar *Insr* mRNA levels to those of mature adipocytes exposed to the same dose (F_int_(3, 32) = 5.482, p = 0.004) (Fig. 6C).

Collectively, these data suggest that early Mn exposure interferes with the programmed metabolic switch from basal to insulin-sensitive glucose transporters, potentially impairing the glucose- handling capacity of the mature adipocytes.

### 3.7. Early manganese exposure during differentiation persistently reduces FABP4 protein levels and alters lipid metabolism gene expression in differentiated adipocytes

**Fig. 7.**
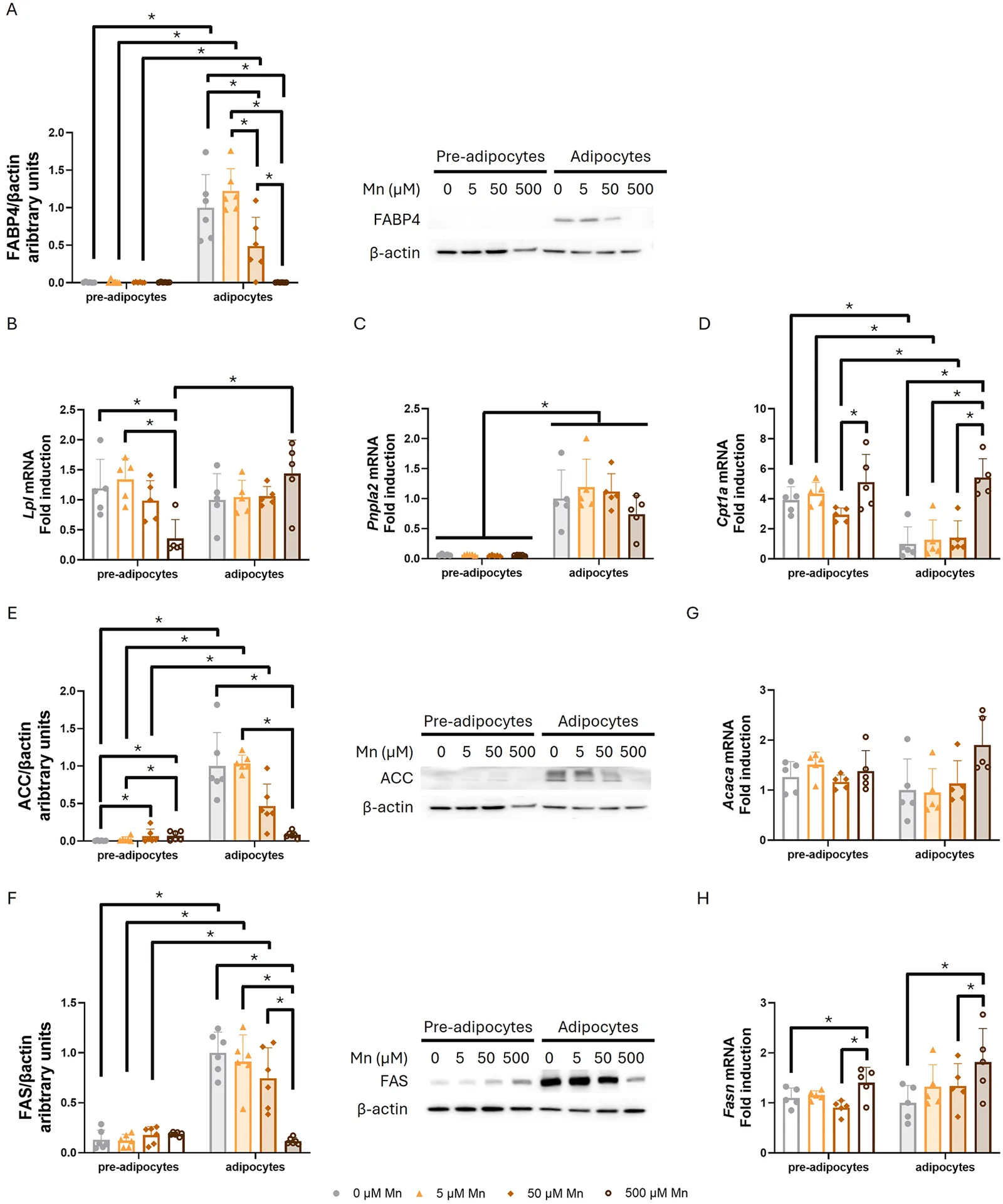
Early Mn exposure during differentiation alters adipocyte lipid metabolism protein and gene expression. Pre-adipocytes were transiently exposed to Mn (0, 5, 50, or 500 μM) during the confluence phase. After Mn removal, cells were either maintained in pre-adipocyte medium or differentiated into mature adipocytes. Protein and mRNA levels of lipid metabolism-related genes were analyzed by western blot and qPCR, respectively. **(A)** FABP4 protein levels quantified by densitometry and representative western blot. **(B)** *Lpl* mRNA expression. **(C)** *Pnpla2* mRNA expression. **(D)** *Cpt1a* mRNA expression. **(E)** ACC protein levels quantified by densitometry and representative western blot. **(F)** FAS protein levels quantified by densitometry and representative western blot. **(G)** *Acaca* mRNA expression. **(H)** *Fasn* mRNA expression. Data are presented as mean ± SD. Statistical significance was determined using two-way ANOVA followed by Bonferroni’s post hoc test. *P* < 0.05 was considered statistically significant. * indicates significant differences. Each point represents an independent biological replicate from a separate cell culture.

Mature adipocytes possess specialized metabolic machinery for lipid uptake, storage, and mobilization. As expected, fully differentiated adipocytes exhibited markedly higher levels of fatty acid-binding protein 4 (FABP4), a cytosolic chaperone that facilitates intracellular fatty acid trafficking and lipid droplet formation. Early Mn exposure significantly decreased FABP4 protein expression in differentiated adipocytes at 50 and 500 μM Mn (F_int_(3, 40) = 16.485, p < 0.001) (Fig. 7A), suggesting a persistent impairment in intracellular lipid handling capacity.

Efficient lipid accumulation in adipocytes also depends on the uptake of circulating lipids. Lipoprotein lipase (LPL) acts as a gatekeeper by hydrolyzing triglycerides, in circulating lipoproteins. Early Mn exposure significantly reduced *Lpl* mRNA levels in pre-adipocytes (F_int_(3, 32) = 6.756, p = 0.001) with a pronounced reduction at the 500 μM concentration (*post hoc*p = 0.009 compared with non-exposed pre-adipocytes). whereas *Lpl* mRNA expression in fully mature adipocytes show no significant changes. Moreover, pre-adipocytes exposed to 500 μM Mn exhibited lower *Lpl* gene expression than fully differentiated adipocytes under the same concentration (*post hoc*p < 0.001) (Fig. 7B).

In contrast to LPL-mediated lipid uptake, the mobilization of intracellular lipid stores is initiated by adipose triglyceride lipase (ATGL), encoded by the p*atatin like phospholipase domain containing 2* (*Pnpla2)* gene. While adipocyte differentiation significantly increased *Pnpla2* gene expression (F_dif_(1, 32) = 115.697, p < 0.001), reflecting an enhanced lipolytic capacity in mature adipocytes, early Mn exposure did not significantly alter *Pnpla2* expression (Fig. 7C).

We also examined carnitine palmitoyltransferase 1A (CPT1a), the rate-limiting enzyme controlling mitochondrial import and oxidation of long-chain fatty acids. As expected, *Cpt1a* gene expression was downregulated during differentiation, reflecting the metabolic switch from fatty acid oxidation to lipid storage. Notably, early exposure to high Mn concentration (500 μM) resulted in a persistent increase in *Cpt1a* mRNA expression in fully differentiated adipocytes (F_int_(3, 32) = 4.564, p = 0.009, *post hoc*p < 0.001 for 500 μM Mn-treated differentiated adipocytes compared with non-exposed differentiated adipocytes) (Fig. 7D). These data suggest that early Mn exposure prevents the physiological decline of fatty acid oxidation, further contributing to reduced lipid accumulation in the differentiated state.

### 3.8. Early manganese exposure differentially regulates lipogenic enzyme expression in pre-differentiated adipocytes

Acetyl-CoA carboxylase (ACC) serves as a critical metabolic switch coordinating lipogenesis and fatty acid oxidation. ACC catalyzes the production of malonyl-CoA, which is both rate-limiting substrate for de novo lipogenesis and a potent inhibitor of CPT1-mediated mitochondrial fatty acid uptake. fatty acid synthase (FAS) subsequently utilizes malonyl-CoA to synthesize palmitate.

As expected, ACC protein levels were significantly higher in differentiated cells than in pre-adipocytes. Notably, early Mn exposure caused distinct, long-lasting effects based on the differentiation status (F_int_(3, 40) = 10.988, p < 0.001). In pre-adipocytes, early Mn exposure persistently increased ACC protein expression, reaching significance at 50 μM (*post hoc*p = 0.035 compared with non-exposed pre-adipocytes) and at 500 μM (*post hoc*p = 0.001 compared with non-exposed pre-adipocytes). In contrast, Mn decreased ACC expression in fully differentiated adipocytes (*post hoc* for 500 μM p = 0.005 compared to non-exposed pre-adipocytes) (Fig. 7E).

Similarly, FAS protein expression increased upon differentiation. While early Mn exposure did not alter FAS levels in pre-adipocytes, it significantly suppressed FAS protein expression in fully differentiated adipocytes at 500 μM Mn (F_int_(3, 40) = 26.112, p < 0.001, *post hoc* for 500 μM p < 0.001 compared with non-exposed pre-adipocytes) (Fig. 7F).

We further assessed the effects of early Mn exposure on the gene expression of *Acaca* and *Fasn*, which encoded ACC and FAS, respectively. While early Mn exposure did not alter *Acaca* mRNA expression (Fig. 7G), it caused a persistent increase in *Fasn* expression at 500 μM Mn (F_Mn_(3, 32) = 4.412, p = 0.01) (Fig. 7H). Interestingly, differentiation status alone did not significantly affect the mRNA expression of either gene, highlighting a clear divergence between transcriptional and translational regulation in this pathway.

Overall, these results suggest that early Mn exposure produces a persistent, differentiation-dependent dysregulation of the ACC-FAS lipogenic pathway, marked by suppressed lipogenic protein expression in fully differentiated adipocytes despite limited transcriptional changes.

### 3.9. Early manganese exposure persistently decreases AMPK and AKT protein levels

**Fig. 8.**
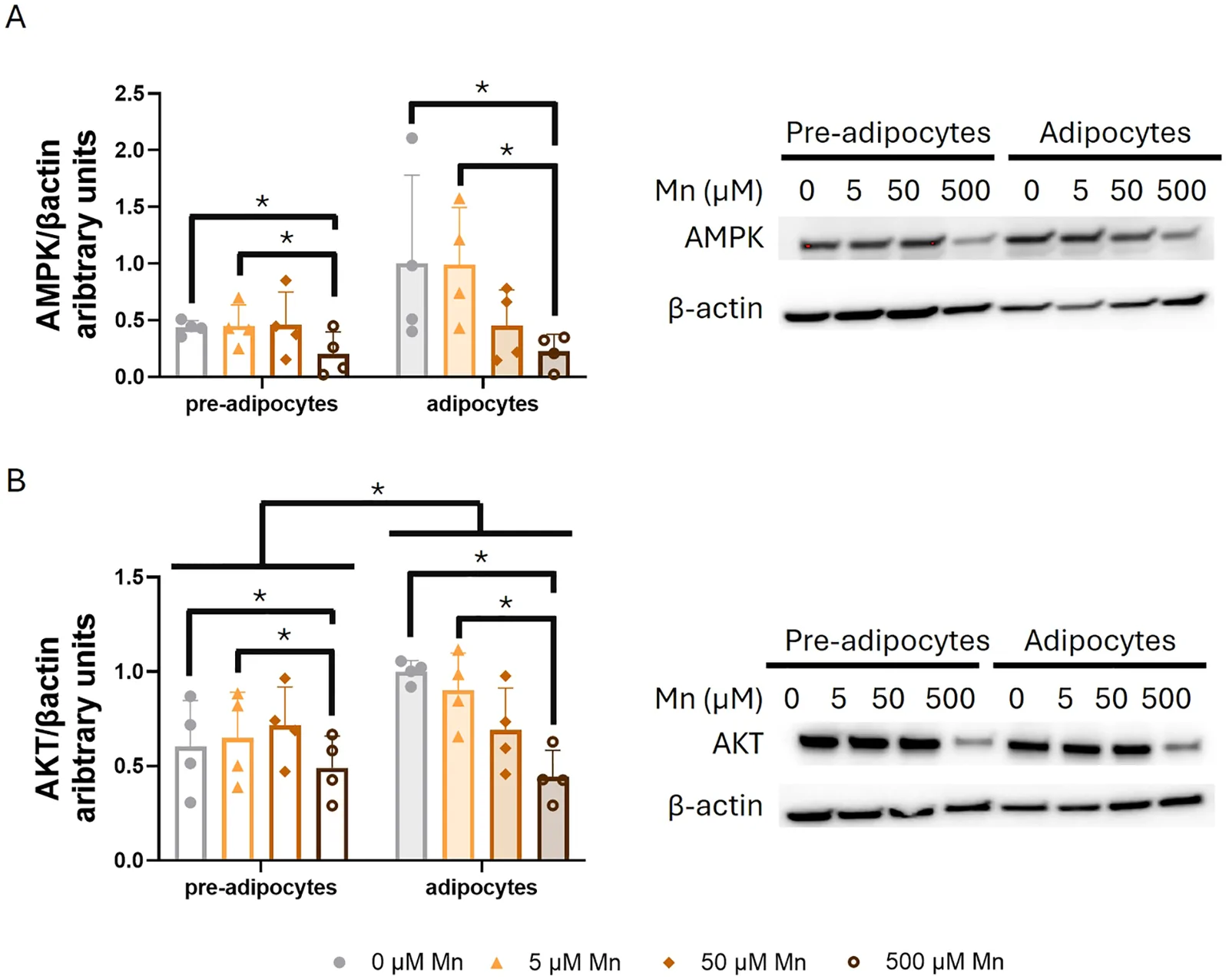
Early Mn exposure during differentiation impairs AMPK and AKT, central regulators of energy homeostasis and insulin signaling. Pre-adipocytes were transiently exposed to Mn (0, 5, 50, or 500 μM) during the confluence phase. After Mn removal, cells were either maintained in pre-adipocyte medium or differentiated into mature adipocytes. Protein expression of metabolic control proteins was analyzed by western blot. **(A)** AMPK protein levels quantified by densitometry and representative western blot. **(B)** AKT protein levels quantified by densitometry and representative western blot. Data are presented as mean ± SD. Statistical significance was determined using two-way ANOVA followed by Bonferroni’s post hoc test. *P* < 0.05 was considered statistically significant. * indicates significant differences. Each point represents an independent biological replicate from a separate cell culture.

AMP-activated protein kinase (AMPK), is a critical energy-sensing kinase that regulates lipid metabolism by phosphorylating and inhibiting ACC, thereby suppressing de novo lipogenesis. In addition, AMPK activity is known to modulate adipogenesis by limiting lipid droplet formation. While differentiation status did not significantly influence AMPK protein levels, early Mn exposure caused a persistent decrease in AMPK protein expression (F_Mn_(3, 24) = 4.969, p = 0.008) (Fig. 8A).

In contrast, protein kinase B (AKT) is a central mediator of insulin-driven anabolic signaling. AKT promotes adipogenesis by enhancing the expression and activity of the adipogenic transcription factors PPARγ and C/EBPα. As expected, AKT protein levels were significantly increased in fully differentiated adipocytes (F_dif_(1, 24) = 4.482, p = 0.045). Notably, early Mn exposure resulted in a persistent reduction of AKT protein expression regardless of the differentiation state (F_Mn_(3, 24) = 4.973, p = 0.008) (Fig. 8B).

Altogether,our data suggest that early Mn exposure induces a persistent reduction of total protein levels of both energy-sensing (AMPK) and insulin-related anabolic signaling (AKT), which may influence adipogenesis and lipid metabolism.

### 3.10. Early manganese exposure persistently impairs thermogenic gene expression in differentiated adipocytes

**Fig. 9.**
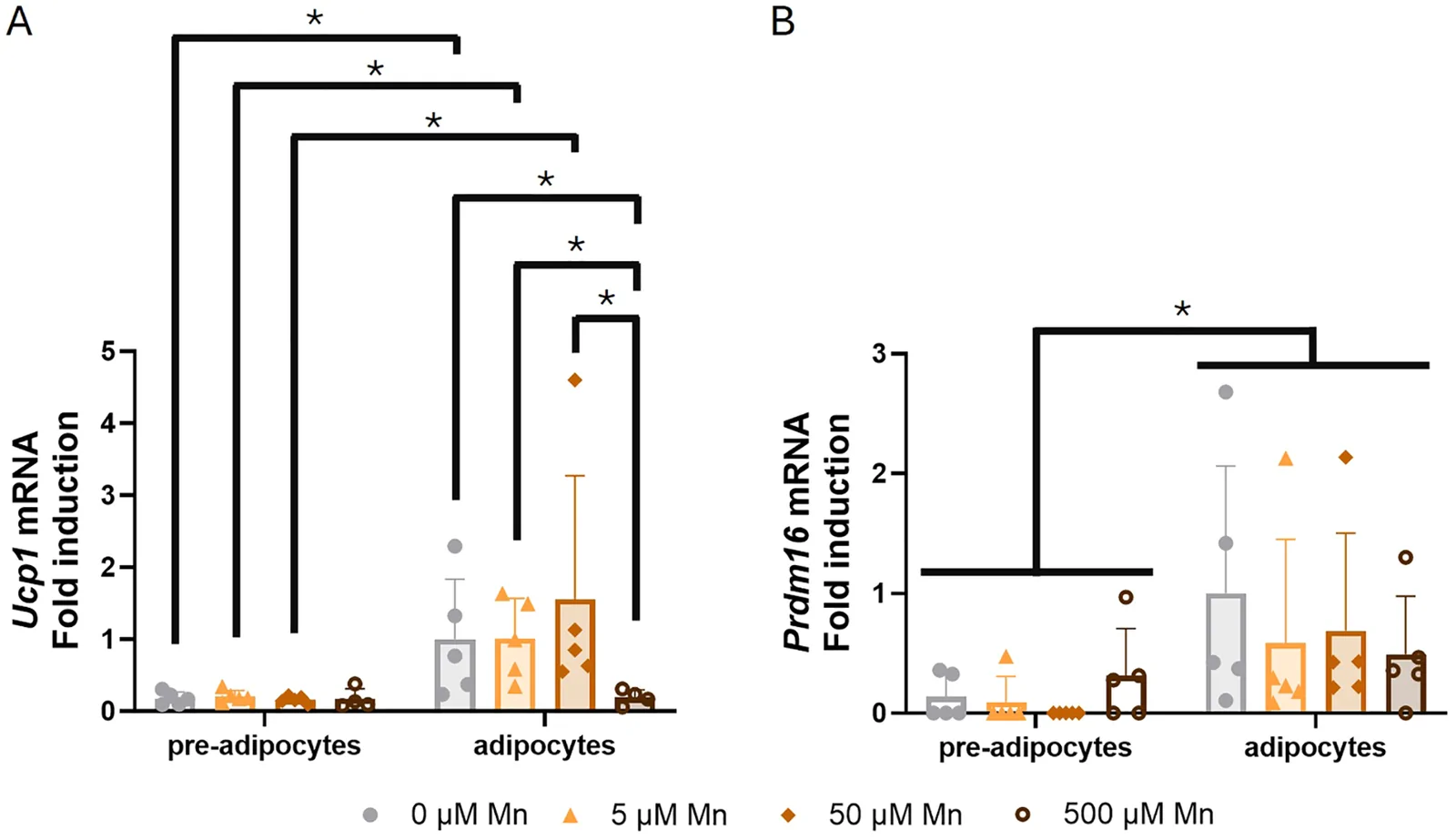
Early Mn exposure during differentiation interferes with the thermogenic program. Pre-adipocytes were transiently exposed to Mn (0, 5, 50, or 500 μM) during the confluence phase. After Mn removal, cells were either maintained in pre-adipocyte medium or differentiated into mature adipocytes. mRNA expression of brown adipocyte genes was analyzed by qPCR. **(A)** *Ucp1* mRNA expression. **(B)** *Prdm16* mRNA expression. Data are presented as mean ± SD. Statistical significance was determined using two-way ANOVA followed by Bonferroni’s post hoc test. *P* < 0.05 was considered statistically significant. * indicates significant differences. Each point represents an independent biological replicate from a separate cell culture.

When 3T3-L1 adipocytes are differentiated using triiodothyronine (T3) and rosiglitazone, the resulting phenotype is a white adipocyte with beige/thermogenic features. A hallmark of this phenotype is the expression of uncoupling protein 1 (UCP1), a mitochondrial inner membrane protein that dissipates the proton gradient to generate heat instead of ATP. As expected, fully differentiated adipocytes exhibited significantly higher *Ucp1* mRNA levels than pre-adipocytes. Notably, early Mn exposure resulted in a persistent reduction of *Ucp1* gene expression in fully differentiated adipocytes (F_int_(3, 30) = 2.985, p = 0.047) (Fig. 9A).

We also assessed the mRNA expression of PR Domain Containing 16 (PRDM16), a key transcriptional co-regulator that drives the brown/beige thermogenic program while repressing white adipocyte-specific pathways. While *Prdm16* gene expression increased significantly upon differentiation, (F_dif_(3, 32) = 8.123, p = 0.008), early Mn exposure had no detectable effect on its mRNA expression (Fig. 9B).

Overall, early Mn exposure persistently suppresses the key thermogenic gene *Ucp1* without altering the expression of its upstream co-regulator gene *Prdm16*. These data suggest that while initial beige/thermogenic programming may be initiated, Mn exposure specifically impairs the execution of the thermogenic program in fully differentiated adipocytes.

### 3.11. Early manganese exposure persistently impairs β-Adrenergic receptor-induced glycerol release in differentiated adipocytes

Having established the impact of early Mn exposure on adipogenic programming, we next examined its persistent effects on stimulated lipolysis. To induce lipolytic activity, pre-adipocytes and fully differentiated adipocytes were treated with either isoproterenol (ISO), a non-selective β-adrenergic receptor agonist, or CL316,243 (CL), a selective β_3_- adrenergic receptor agonist. Glycerol release into the culture medium was measured 30 min post-stimulation as a direct indicator of activated lipolysis.

**Fig. 10.**
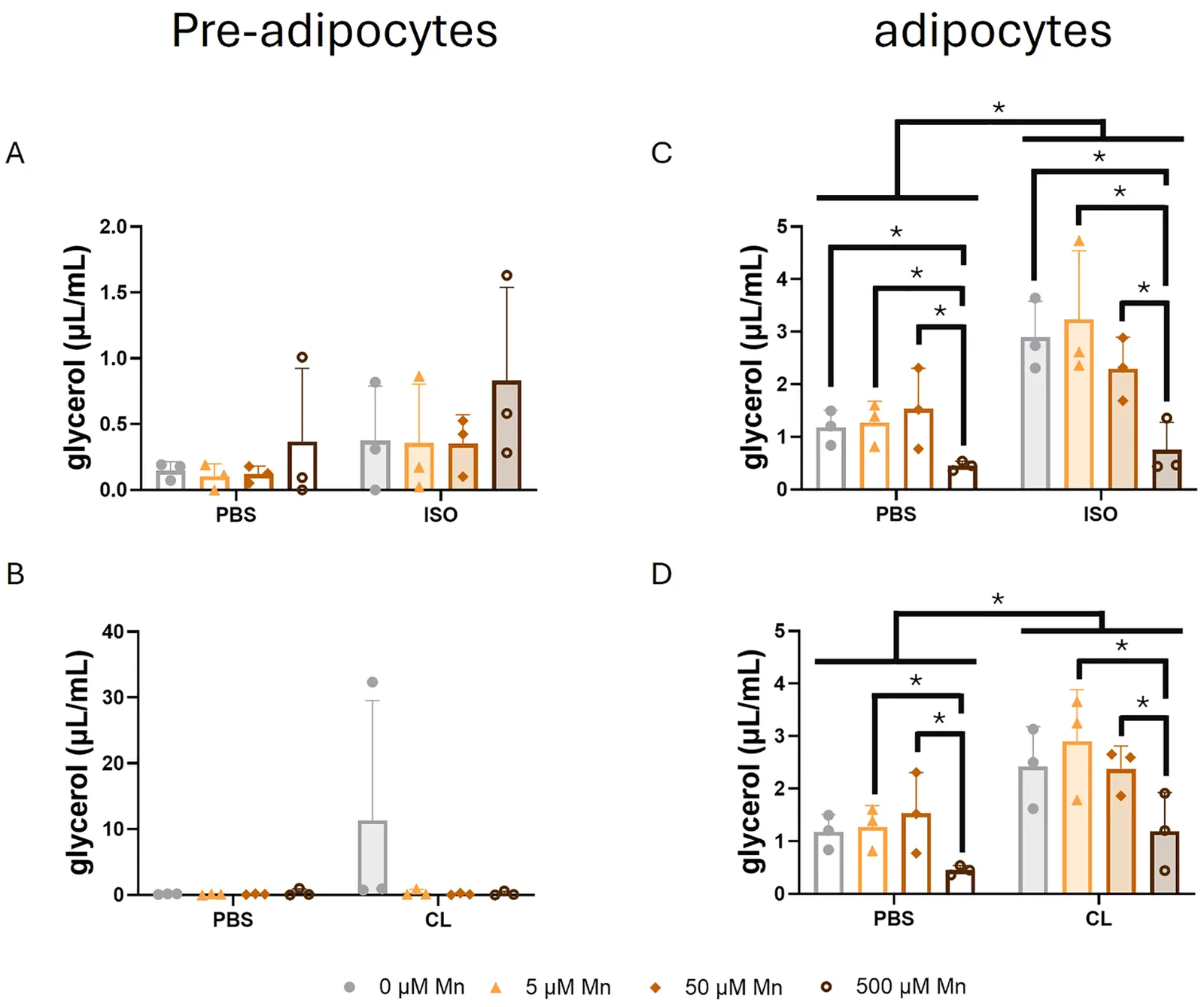
Early Mn exposure persistently impairs induced lipolysis in fully differentiated adipocytes. Transiently Mn exposed (0, 5, 50, or 500 μM) pre-adipocytes and fully differentiated adipocytes were treated with 10 μM of non-selective β-adrenergic receptor agonist isoproterenol (ISO) or with 10 μM of β3-adrenergic receptor agonist CL 316,243 (CL) for 30 min in a humidified incubator at 37 °C with 5% CO_2_. Following treatment, glycerol release into the medium was measured.**(A)** ISO-induced glycerol release in pre-adipocytes. **(B)** CL-induced glycerol release in pre-adipocytes. **(C)** ISO-induced glycerol release in fully differentiated adipocytes. **(D)** CL-induced glycerol release in fully differentiated adipocytes. Data are presented as mean ± SD. Statistical significance was determined using two-way ANOVA followed by Bonferroni’s post hoc test. *P* < 0.05 was considered statistically significant. * indicates significant differences. Each point represents an independent biological replicate from a separate cell culture.

In pre-adipocytes, no significant effects were detected following treatment with either ISO (Fig. 10A) or CL (Fig. 10B), consistently with their lack of mature adipocyte machinery. As expected, ISO significantly stimulated glycerol release in fully differentiated adipocytes (F_ago_(1, 16) = 18.458, p < 0.001) (Fig. 10C). Notably, early Mn exposure significantly attenuated this response at 500 μM Mn (F_Mn_(3, 16) = 7.318, p = 0.003, *post hoc* for 500 μM p = 0.012 compared with non-Mn exposed adipocytes) (Fig. 10C).

A similar impairment was observed when lipolysis was induced with β_3_-selective agonist CL (F_ago_(1, 16) = 18.771, p < 0.001) (Fig. 10D). Early Mn exposure persistently reduced the lipolytic output in these cells (F_Mn_(3, 16) = 5.051, p = 0.012) (Fig. 10D). Altogether, these data indicate that exposure during the confluency phase induces a long-lasting deficit in the cell’s ability to mobilize lipid stores in response to β-adrenergic stimulation.

### 3.12. Early manganese exposure persistently impairs lipogenic protein upregulation during stimulated lipolysis in differentiated adipocytes

**Fig. 11.**
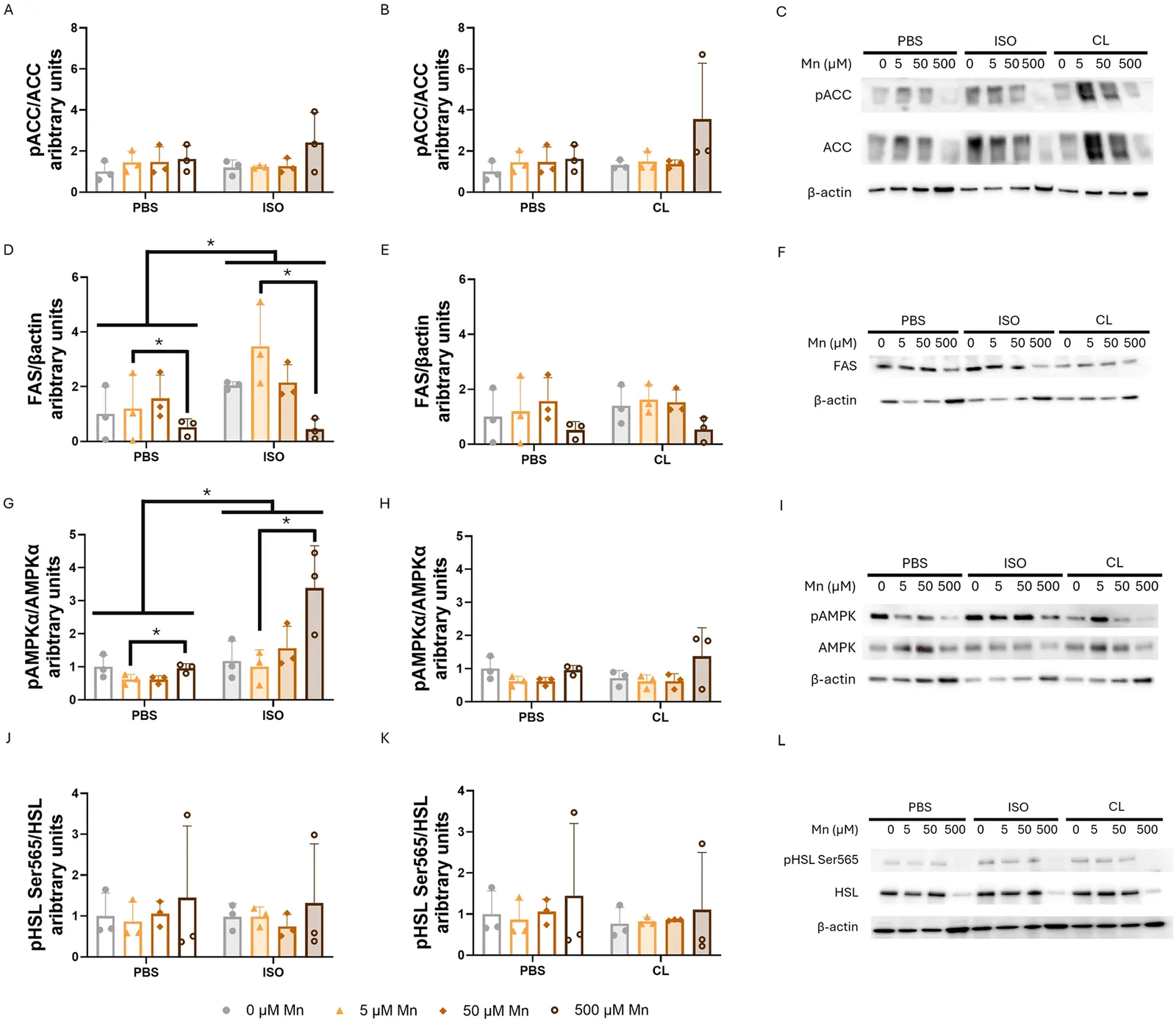
Transient Mn exposure during differentiation persistently impairs lipogenic program and β-adrenergic-AMPK-HSL signaling in fully differentiated adipocytes. Transiently Mn exposed (0, 5, 50, or 500 μM) mature adipocytes were treated with 10 μM of non-selective β-adrenergic receptor agonist isoproterenol (ISO) or with 10 μM of β3-adrenergic receptor agonist CL 316,243 (CL) for 30 min in a humidified incubator at 37 °C with 5% CO_2_. Following treatment, protein expression levels of lipogenic proteins were analyzed by western blot. **(A)** Phospho-ACC/total ACC ratio quantified by densitometry in adipocytes treated with ISO. **(B)** Phospho-ACC/total ACC ratio quantified by densitometry in adipocytes treated with CL. **(C)** Representative western blot of phospho-ACC, ACC, and β-actin. **(D)** FAS quantified by densitometry in adipocytes treated with ISO. **(E)** FAS quantified by densitometry in adipocytes treated with CL. **(F)** Representative western blot of FAS and β-actin. **(G)** Phospho-AMPKα/total AMPKα ratio quantified by densitometry in adipocytes treated with ISO. **(H)** Phospho-AMPKα/total AMPKα ratio quantified by densitometry in adipocytes treated with CL. **(I)** Representative western blot of phospho-AMPKα, AMPKα, and β-actin. **(J)** Phospho-HSL Ser565/total HSL ratio quantified by densitometry in adipocytes treated with ISO. **(K)** Phospho-HSL Ser565/total HSL ratio quantified by densitometry in adipocytes treated with CL. **(L)** Representative western blot of phospho-HSL Ser565, HSL, and β-actin. Data are presented as mean ± SD. Statistical significance was determined using two-way ANOVA followed by Bonferroni’s post hoc test. *P* < 0.05 was considered statistically significant. * indicates significant differences. Each point represents an independent biological replicate from a separate cell culture.

Given the reduction in glycerol release, we investigated the signaling mechanisms regulating lipid metabolism in fully differentiated adipocytes under stimulated lipolysis. We first examined the response of lipogenic enzymes. Following ISO stimulation, no inhibition of ACC was observed, as evidenced by the stable phospho-ACC/ACC ratio (Fig. 11A). However, we observed significant interaction regarding total ACC protein levels (F_int_(3, 16) = 6.245, p = 0.005) (Supp. Fig. 1A). Surprisingly, ISO stimulation increased total ACC protein levels, an effect that was ameliorated in adipocytes previously exposed to 500 μM Mn. Similarly, phospho-ACC levels were increased following ISO treatment (F_ago_(1, 16) = 10.060, p = 0.006) (Supp. Fig. 1B) but were significantly reduced by early Mn exposure (F_Mn_(3, 16) = 11.390, p < 0.001) (Supp. Fig. 1B).

A similar pattern emerged under β_3_-adrenergic stimulation with CL. Although the phospho-ACC/ACC ratio remained unchanged (Fig. 11B), CL stimulation increased both total ACC (F_ago_(1, 16) = 9.881, p = 0.006) (Supp. Fig. 1C), and phospho-ACC protein levels (F_ago_(1, 16) = 8.797, p = 0.009) (Supp. Fig. 1D). Early Mn exposure persistently decreased both total ACC (F_Mn_(3, 16) = 12.683, p < 0.001) (Supp. Fig. 1C) and phospho-ACC protein expression (F_Mn_(3, 16) = 11.151, p < 0.001) (Supp. Fig. 1D). The observation that protein levels increased while the phosphorylation ratio remain stable suggests that, at 30 min post-stimulation, lipogenic pathways are not fully inhibited by the adrenergic signal.

Next, we examined FAS protein levels. Stimulation with the non-selective agonist ISO induced a significant increase in FAS protein levels (F_ago_(1, 16) = 7.034, p = 0.017) (Fig. 11D), whereas treatment with the selective β_3_ agonist CL did not produce significant changes (Fig. 11E). Notably, early Mn exposure decreased FAS protein levels, particularly in ISO-treated cells (F_Mn_(3, 16) = 4.782, p = 0.015) (Fig. 11D). These data suggest that the upregulation of FAS in response to β-adrenergic stimulation is mediated through β_3_-adrenergic receptor-independent mechanisms and is persistently compromised by early Mn exposure.

### 3.13. Early manganese exposure persistently disrupts β-adrenergic-AMPK-HSL signaling and attenuates lipolysis in differentiated adipocytes

We next investigated whether the impaired lipolysis was linked to dysregulation of the AMPK and hormone-sensitive lipase (HSL) signaling axis. Inhibition of the lipogenic enzyme ACC is typically mediated by the energy sensor AMPK. ISO treatment significantly increased the phospho-AMPKα/AMPKα ratio (F_ago_(1, 16) = 16.843, p < 0.001) (Fig. 11G), whereas CL stimulation had no effect (Fig. 11H). Notably, early exposure to 500 μM Mn persistently further increased the phospho-AMPKα/AMPKα ratio, in ISO-treated cells (F_Mn_(3, 16) = 5.101, p = 0.011) (Fig. 11G). In parallel, Mn exposure significantly reduced total AMPKα (F_Mn_(3, 16) = 9.571, p < 0.001 (Supp. Fig. 2A) and F_Mn_(3, 16) = 11.942, p < 0.001 (Supp. Fig. 2C)) and phospho-AMPKα levels ((F_Mn_(3, 16) = 5.312, p = 0.010 (Supp. Fig. 2B) and F_Mn_(3, 16) = 8.481, p = 0.001 (Supp. Fig. 2D)) across both ISO and CL conditions.

AMPK can negatively regulate lipolysis by phosphorylating HSL at Ser565, a site that prevents activation by PKA. Under our conditions, the phospho-HSL(Ser565)/HSL ratio was not altered by ISO (Fig. 11J) or CL (Fig. 11K), suggesting this inhibitory pathway was not engaged. However, early Mn exposure led to a persistent and significant reduction in both total HSL (F_Mn_(3, 16) = 13.376, p < 0.001 (Supp. Fig. 2E) and F_Mn_(3, 16) = 19.905, p < 0.001 (Supp. Fig. 2 G)) and phospho-HSL (Ser565) levels (F_Mn_(3, 16) = 9.416, p < 0.001 (Supp. Fig. 2F) and F_Mn_ (3, 16) = 10.801, p < 0.001 (Supp. Fig. 2H)) following ISO and CL stimulation.

**Fig. 12.**
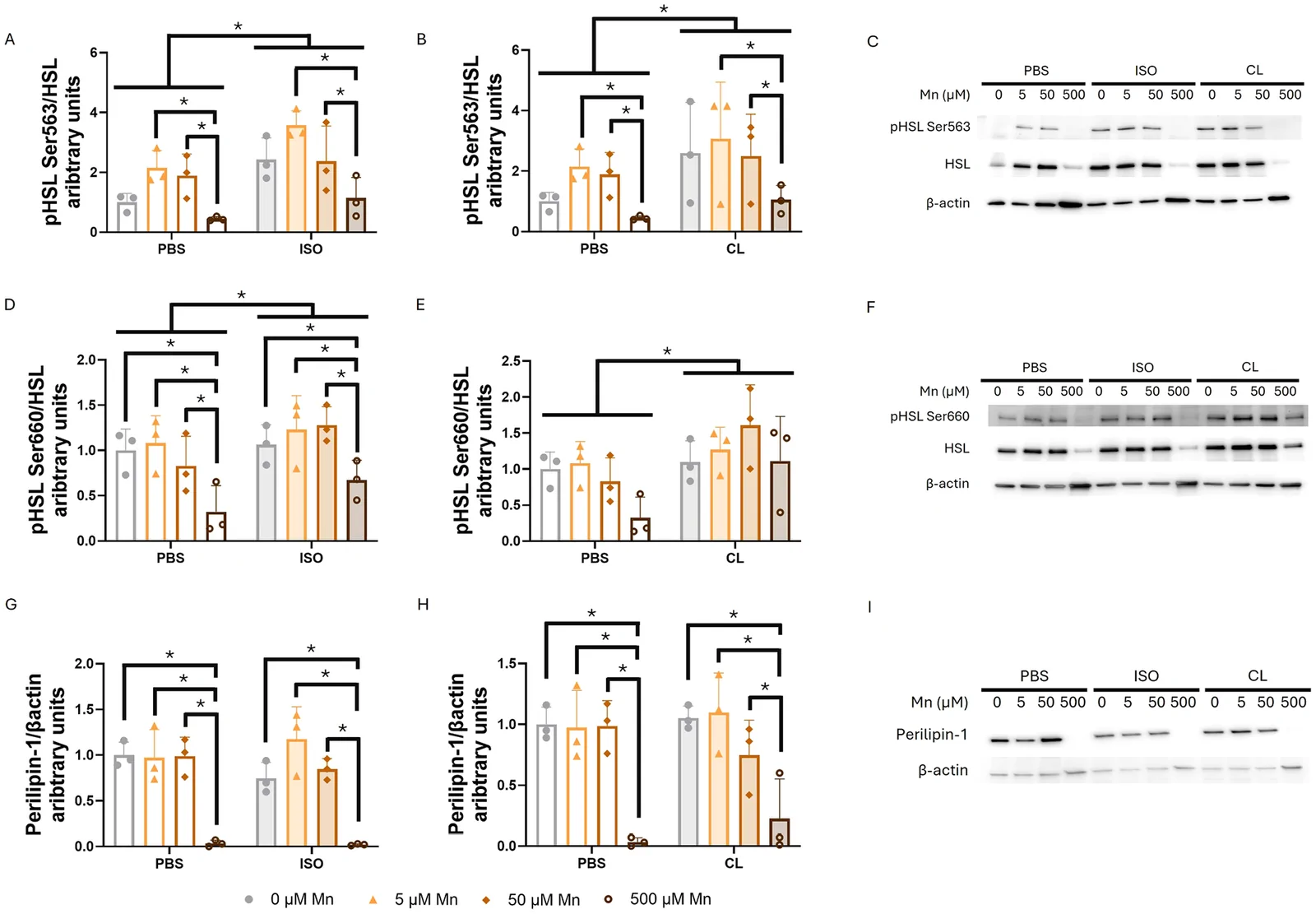
Early Mn exposure during differentiation persistently impairs β-adrenergic-HSL phosphorylation and Perilipin-1 protein levels in fully differentiated adipocytes. Transiently Mn exposed (0, 5, 50, or 500 μM) mature adipocytes were treated with 10 μM of non-selective β-adrenergic receptor agonist isoproterenol (ISO) or with 10 μM of β3-adrenergic receptor agonist CL 316,243 (CL) for 30 min in a humidified incubator at 37 °C with 5% CO_2_. Following treatment, protein phosphorylation of HSL was analyzed by western blot. **(A)** Phospho-HSL Ser563/total HSL ratio quantified by densitometry in adipocytes treated with ISO. **(B)** Phospho-HSL Ser563/total HSL ratio quantified by densitometry in adipocytes treated with CL. **(C)** Representative western blot of phospho-HSL Ser563, HSL, and β-actin. **(D)** Phospho-HSL Ser660/total HSL ratio quantified by densitometry in adipocytes treated with ISO. **(E)** Phospho-HSL Ser660/total HSL ratio quantified by densitometry in adipocytes treated with CL. **(F)** Representative western blot of phospho-HSL Ser660, HSL, and β-actin. **(G)** Perilipin-1 quantified by densitometry in adipocytes treated with ISO. **(H)** Perilipin-1 quantified by densitometry in adipocytes treated with CL. **(I)** Representative western blot of perilipin-1, and β-actin. Data are presented as mean ± SD. Statistical significance was determined using two-way ANOVA followed by Bonferroni’s post hoc test. *P* < 0.05 was considered statistically significant. * indicates significant differences. Each point represents an independent biological replicate from a separate cell culture.

To evaluate lipolytic activation, we examined PKA-mediated phosphorylation of HSL at Ser563 and Ser660, which enhances catalytic activity and promotes translocation to lipid droplet. As expected, ISO (F_ago_(1, 16) = 13.673, p = 0.002) (Fig. 12A) and CL (F_ago_(1, 16) = 4.553, p = 0.049) (Fig. 12B) increased the phospho-HSL(Ser563)/HSL ratio. Notably, early Mn exposure significantly attenuated this activation (F_Mn_(3, 16) = 10.012, p < 0.001 (Fig. 12A) and F_Mn_(3, 16) = 5.857, p = 0.007 (Fig. 12B)). While the agonist did not alter total HSL protein levels, early Mn exposure significantly decreased HSL protein expression (F_Mn_(3, 16) = 7.623, p = 0.002 (Supp. Fig. 3B) and F_Mn_(3, 16) = 6.432, p = 0.005 (Supp. Fig. 3D)). A similar trend was observed for the Ser660 site: ISO significantly increased the phospho-HSL(Ser 660)/HSL ratio (F_ago_(1, 16) = 5.024, p = 0.040) (Fig. 12D), an effect attenuated by early Mn exposure (F_Mn_(3, 16) = 6.851, p = 0.004) (Fig. 12D). CL similarly increased the phospho-HSL(Ser660)/HSL ratio (F_ago_(1, 16) = 8.398, p = 0.010) (Fig. 12E); however, early Mn exposure did not significantly affect this response.

Finally, we analyzed perilipin-1, the lipid droplet-associated protein essential for HSL docking and efficient triglyceride breakdown. Early exposure to 500 μM Mn caused a persistently decreased perilipin-1 protein levels under both conditions (F_Mn_(3, 16) = 32.283, p < 0.001 (Fig. 12G) and F_Mn_(3, 16) = 18.985, p < 0.001 (Fig. 12H)).

Collectively, these findings indicate that early Mn exposure induces a multi-level failure of the lipolytic machinery, characterized by reduced HSL activation, lower HSL protein availability, and the loss of the structural protein perilipin-1.

## 4. Discussion

The increasing burden of metabolic disorders has been linked to environmental exposures that interfere with endocrine and metabolic regulation. In this novel study, we demonstrate that Mn exposure during the pre-adipocyte confluence phase induces a long-lasting concentration-related disruption of adipogenic programming, resulting in an impairment of adipocyte maturation. Our findings suggest that early Mn exposure interferes with the molecular machinery required for adipocyte function. Importantly, the inclusion of both lower and higher Mn concentrations allowed us to distinguish potential lower-dose programming effects from high-dose toxicity-associated effects. Lower Mn concentrations may reflect persistent effects on adipocyte maturation, whereas higher concentrations likely involve substantial cellular stress or toxicity.

Human population data suggests a relationship between Mn status and visceral adiposity(Tao, Huang et al., 2022), supporting the relevance of Mn exposure to adipose tissue biology. However, available tissue-distribution studies indicate that Mn accumulation in adipose tissue is relatively limited compared with other organs, even under high-exposure conditions. In rats exposed orally to MnCl_2_ at 75 mg Mn/kg/day for 4 weeks, adipose tissue Mn levels reached only 0.14 ± 0.07 μg/g wet weight and were not significantly different from controls (Missy, Lanhers et al., 2000). Similarly, intravenous MnCl_2_ studies in mice showed preferential Mn accumulation in the pancreas, kidney, liver, and heart, whereas fat/adipose tissue showed low or negligible accumulation (Lee, So et al., 2010). Importantly, limited Mn accumulation in adipose tissue does not preclude biological effects at lower concentrations or during sensitive windows of adipocyte development. In this context, it remains relevant to determine whether transient Mn exposure during the early stages of adipocyte differentiation can produce persistent alterations in adipocyte maturation and metabolic function.

Both deficient and excessive Mn levels are associated with pathological dysfunctions (Martins, Oliveira-Paula et al., 2025). Specifically, excessive Mn exposure is a well-established cause of parkinsonism, a clinical syndrome characterized by motor dysfunctions arising from altered dopaminergic signaling (Lucchini and Tieu, 2023). In recent years, increasing attention has been directed toward the role of Mn in metabolic diseases (Li and Yang, 2018; Baj, Flieger et al., 2023); however, despite growing interest, epidemiological and experimental findings remain inconsistent, underscoring the need for further mechanistic investigation.

In our study, a transient 48-hour Mn exposure during the confluent phase resulted in a persistent reduction in lipid accumulation, even after Mn removal. This decrease in lipid content was accompanied by a downregulation in PPARγ and C/EBPα protein expression. These transcription factors play a central role in adipogenesis, while PPARγ functions as the master regulator of the adipocyte development, C/EBPα is required for terminal differentiation and full adipocyte maturation. (Moseti, Regassa et al., 2016; Ghaben and Scherer, 2019). Given that adipocyte differentiation follows a tightly regulated transcriptional hierarchy, early Mn exposure could compromise adipogenesis by interfering with PPARγ- and C/EBPα-mediated signaling, thereby reducing lipid accumulation. Data from the highest Mn concentration should be interpreted carefully, as 500 μM Mn also persistently reduced MTT metabolic activity. Therefore, the most pronounced effects observed at this concentration may reflect impaired cell health and cellular stress, in addition to any specific effects on adipogenic signaling. A limitation of the present study is that acute cytotoxicity was not assessed immediately after the 48-hour Mn exposure. Consequently, early cellular stress or damage cannot be excluded as a contributor to the persistent alterations observed after differentiation, particularly at 500 μM Mn. Further studies specifically designed to characterize acute cytotoxicity and concentration-response relationships will be required to distinguish direct effects on adipogenic programming from consequences of early cellular injury.

Although early Mn exposure decreased PPARγ protein levels, these effects were not reflected at the transcriptional level, as *Pparg* mRNA expression remained unchanged. This dissociation between protein and mRNA levels suggests that early Mn exposure could affect protein stability, translation efficiency, or turnover rather than gene transcription. Recent studies have demonstrated that even brief mitochondrial stress can remodel the nascent proteome independent of mRNA levels, largely through dynamic regulation of elongation factors such as EEF1A1, whose downregulation persists beyond stress relief (Stepkowski, Linke et al., 2024). Early Mn exposure during initial steps of differentiation may act as a stressor that similarly modulates translation, contributing to reduced PPARγ protein accumulation despite unchanged *Pparg* mRNA. In addition, oxidative and mitochondrial stress can disrupt protein homeostasis by promoting oxidative protein modifications and activating protein degradation pathways, including the ubiquitin–proteasome and autophagy–lysosomal systems (Pajares, Jimenez-Moreno et al., 2015). This possibility is supported by studies showing that Mn can dysregulate autophagy and lysosomal function in neural models (Zhang, Cao et al., 2013; Zhang, Liu et al., 2025), and that oxidative stress can reduce adiponectin protein abundance in adipocytes through ubiquitin–proteasome-mediated degradation despite increased gene expression (Wang, Dou et al., 2012). Moreover, proteasome dysfunction has been reported to impair adipogenesis, suggesting that proteasome-dependent protein turnover is required for normal adipocyte maturation (Willemsen, Arigoni et al., 2022). Thus, Mn-induced disruption of translation and/or protein turnover could contribute to reduced adipogenic protein levels despite unchanged or increased mRNA expression. Further studies are needed to clarify the precise post-transcriptional mechanisms involved and their effects on adipocyte function.

Terminal adipocyte differentiation is ultimately driven by the expression of genes involved in lipid uptake, triglyceride synthesis, and insulin responsiveness (Moseti, Regassa et al., 2016). In this context, early Mn exposure, particularly at higher concentrations, disrupted the lipid-handling machinery in mature adipocytes, as evidenced by decreased protein levels of ACC and FAS, which are involved in de novo lipogenesis, FABP4, a chaperone that mediates fatty acid transport, and perilipin-1, which stabilizes lipid storage and limits basal lipolysis. Under physiological conditions, the presence of these proteins indicates successful maturation and metabolic competence. Consistent with this, Mn exposure during the early stages of differentiation was associated with reduced expression of these markers at later stages. Together with the observed decreases in PPARγ and C/EBPα, these findings suggest that early Mn exposure may impair the development of fully functional adipocytes.

Upon reaching terminal maturation, adipocytes acquire the capacity to secrete adipokines, a heterogeneous group of signaling molecules that exert pleiotropic effects on lipid metabolism, insulin responsiveness, and inflammation (Taylor, 2021; Pestel, Blangero et al., 2023). The long-lasting effects of early Mn exposure observed in the adipokines adiponectin and leptin were consistent with the broader impairments we observed in adipogenesis. Adiponectin and leptin are hallmark markers of mature adipocytes, and their expression depends on the coordinated activation of adipogenic transcription factors PPARγ and C/EBPα (Maeda, Takahashi et al., 2001; Gustafson, Jack et al., 2003; Yang, Brown et al., 2004; Qiao, Maclean et al., 2005; Koshiishi, Park et al., 2008). The persistent reduction in adiponectin, combined with the decline in *leptin* mRNA expression observed mainly at the highest Mn concentration, suggests that Mn-exposed cells fail to reach full metabolic maturity and persistently disrupt the endocrine profile, leading to a significant loss of their functional identity. Interestingly, *leptin* gene expression exhibited stage-dependent responses to early Mn exposure, with upregulation in pre-adipocytes and suppression in mature adipocytes, suggesting that early Mn exposure differentially alters the transcriptional environment and generates a “priming effect” in which an acute Mn exposure may ultimately limit the cell’s adipogenic capacity. The compensatory upregulation of the *leptin receptor* gene expression observed in both stages likely reflects a cellular effort to maintain signaling sensitivity despite declining leptin expression. Early Mn exposure led to persistent changes in adipokine expression, suggesting that it could compromise the adipocyte endocrine function. Overall, these adipokine-related findings should be interpreted in a concentration-dependent manner, with the clearest alterations observed at 500 μM Mn, whereas lower concentrations produced more limited or non-significant effects.

Adipocyte differentiation involves a metabolic transition from predominantly GLUT1-mediated basal glucose uptake toward GLUT4-dependent, insulin-stimulated glucose uptake (Hauner, Rohrig et al., 1998). During normal adipocyte differentiation, *Slc2a1* mRNA expression declines as cells transition from basal, insulin-independent glucose uptake toward GLUT4-mediated insulin responsiveness. Consistent with this, unexposed differentiated adipocytes exhibited lower *Slc2a1* expression compared to pre-adipocytes. Early Mn exposure reduced *Slc2a1* expression in pre-adipocytes, suggesting a potential impairment of basal glucose uptake in these cells. In contrast, early Mn-exposed differentiated adipocytes maintained *Slc2a1* expression at levels analogous to unexposed pre-adipocytes. Notably, these mature adipocytes also exhibited a reduction in *Slc2a4* mRNA expression, indicating a disruption of the normal GLUT1-to-GLUT4 transition. Thus, early Mn exposure may disrupt the normal developmental regulation of glucose transporters, leading to persistent alteration in glucose handling that favors insulin-independent uptake in mature adipocytes.

Notably, early Mn exposure exhibited a persistent dose-dependent modulation of *Slc2a4* mRNA expression, with low concentrations enhancing its gene expression and higher concentrations resulting in a marked reduction. This biphasic response is consistent with evidence that inadequate and excessive Mn availability can dysregulate glucose handling, potentially leading to the development of metabolic syndrome (Shan, Chen et al., 2016; Li and Yang, 2018; Sun, Shao et al., 2023). Epidemiological studies have shown that Mn deficiency is associated with higher fasting glucose levels (Soto-Sanchez, Hernandez-Mendoza et al., 2025) and an increased prevalence of diabetes (Koh, Kim et al., 2014). Similarly, *in vivo* studies indicate that Mn deficiency impairs glucose homeostasis (Baly, Schneiderman et al., 1990) and lipid metabolism (Hu, Tang et al., 2025), effects that can be reversed by Mn supplementation (Hu, Tang et al., 2025). Mn treatment also appears to improve glucose homeostasis by reducing diabetes-related biomarkers in diabetic rats (Burlet and Jain, 2017) and improving insulin secretion and glucose tolerance under conditions of dietary stress (Lee, Jouihan et al., 2013). In our study, the lower Mn concentration may reflect these beneficial effects. At the highest concentration that we tested, the transition from therapeutic to toxic effects resulted in decreased *Slc2a4* gene expression and suppressed adipocyte maturation. These data highlight the importance of maintaining appropriate Mn levels to preserve insulin-stimulated glucose uptake and adipocyte maturation.

GLUT1 and GLUT4 play important roles in mediating glucose uptake in brown adipocytes, supporting the energetic demands of thermogenic activity (McNeill, Morton et al., 2020). However, the effects of Mn exposure on brown/beige adipocytes function remain poorly understood. Although 3T3-L1 cells are most commonly used as a white adipocyte model, they have also been used to investigate beige or brown-like differentiation when subjected to specific adipogenic stimuli (Karamanlidis, Karamitri et al., 2007; Aziz, Wakeling et al., 2017; Lone, Parray et al., 2018; Nueda, Gonzalez-Gomez et al., 2018; Seo, Jin et al., 2019; Manigandan, Mukherjee et al., 2021; Sawamoto, Kanazaki et al., 2021; Patel, Dobbins et al., 2022; Hamzah, Zamri et al., 2025). The differentiation protocol used in this study was designed to generate white adipocyte with brown/beige adipocytes characteristics (Asano, Kanamori et al., 2014). This methodological choice allowed us to examine thermogenic endpoints within the differentiated adipocyte model. Our data confirmed the expression of brown/beige adipocyte markers. Although *Prdm16* gene expression remained unchanged, early Mn exposure downregulated *Ucp1* mRNA levels, suggesting that Mn exposure persistently affects late stage of differentiation rather than initial lineage specification. This dissociation may result from Mn-induced oxidative stress or altered signaling pathways that specifically modulates UCP1 expression. Early Mn exposure may compromise the adipocytes capacity for thermogenesis and energy expenditure, which could have broader consequences for metabolic homeostasis.

β-adrenergic-stimulation promotes lipolysis through site-specific activation of HSL. Both ISO and CL induced glycerol release in fully differentiated adipocytes, confirming that the observed activation of HSL translated into enhanced lipolysis. In this study, early Mn exposure was associated with reduced glycerol release and decreased phosphorylation of HSL at Ser563 and Ser660 in fully differentiated adipocytes, suggesting that Mn exposure during early stages of differentiation is sufficient to exert long-lasting inhibitory effects on the cell’s lipolytic capacity. Our results are consistent with previous reports demonstrating that Mn reduces glycerol release *in vitro* and *in vivo* (Rennie and Upton, 1981; Baquer, Sinclair et al., 2003). Mechanistically, it has been proposed that Mn impairs ISO-induced glycerol release by diminishing the cAMP response, thereby interfering with the mobilization of store triglycerides into free fatty acids and glycerol during lipolysis (Rennie and Upton, 1981). The β-adrenergic agonists unexpectedly did not fully suppress the de novo lipogenesis signaling pathway, an effect especially evident when the cells were stimulated with the non-selective β-adrenergic agonist (ISO). Interestingly, activation of de novo lipogenesis following β-adrenergic stimulation has been also observed *in vivo* after chronic treatment with β-adrenergic agonists (Mottillo, Balasubramanian et al., 2014; Buzelle, MacPherson et al., 2015; Guilherme, Yenilmez et al., 2020; Queathem, Welly et al., 2021).

Excessive Mn accumulation is known to induce mitochondrial dysfunction and oxidative stress (Lu, Deng et al., 2023; Martins, Oliveira-Paula et al., 2025). In the present study, transient Mn exposure followed by prolonged Mn withdrawal resulted in a persistent reduction in SOD2 protein in pre-adipocytes, without a compensatory Nrf2 gene response, indicating a stable, non-transcriptional reprogramming of mitochondrial redox homeostasis. However, in differentiated adipocytes a post-transcriptional decoupling emerged. While early Mn-exposed adipocytes upregulated antioxidant Nrf2-target genes like *Hmox1, Slc7a11*, and *Nqo1*, they remained unable to restore HO-1 and SOD2 protein levels. As we previously mentioned, the dissociation between antioxidant proteins and mRNA levels suggests that Mn may affect post-transcriptional regulation or protein stability rather than gene transcription per se, consistent with a persistent imprint of early oxidative stress on the adipose antioxidant response. Altogether, these findings suggest that early Mn exposure produces long-lasting, differentiation stage-dependent alterations in antioxidant machinery.

Published studies have shown that sustained induction of HO-1 impairs adipocyte differentiation (Wagner, Lindroos-Christensen et al., 2017; Suryaningtyas, Lee et al., 2024). Based on this, one possible hypothesis is that Mn exposure prior to differentiation may transiently elevate intracellular ROS, leading to increased HO-1 expression at early stages, which could contribute to an impaired adipogenic program. Although the present study did not directly assess early ROS production or HO-1 induction prior to differentiation, this hypothesis is consistent with the capacity of Mn to induce HO-1 protein expression ((Li, Wu et al., 2011; Bahar, Kim et al., 2017; Gorojod, Alaimo et al., 2018) and the reported inhibitory effects of HO-1 on adipocyte differentiation (Wagner, Lindroos-Christensen et al., 2017). However, the directionality of the HO-1 response remains unclear. The reduced HO-1 protein levels observed after differentiation may reflect incomplete adipocyte maturation or altered protein turnover. Further studies are needed to clarify whether Mn-driven oxidative stress and HO-1 signaling directly disrupt adipocyte maturation or occur secondarily as a consequence of impaired differentiation.

Overall, prior early Mn exposure showed concentration-dependent effects on adipocyte differentiation and metabolic function. At the intermediate concentration (50 μM), early Mn exposure resulted in reduced lipid accumulation, and the downregulation of key adipogenic transcription factors, suggesting a disruption of early adipocyte differentiation in the absence of the strongest toxicity signal. In contrast, the highest concentration (500 μM) led to global suppression of adipogenic program blocking the establishment of the adipocyte identity. These findings suggest that brief Mn exposure during the initial stages of the differentiation protocol can have lasting effects on adipocyte maturation at lower concentrations, in the absence of overt toxicity. However, the effects observed at the highest Mn concentration should be interpreted with caution, as they may reflect generalized cellular stress or toxicity rather than specific alterations in the adipogenic program alone.

## 5. Conclusion

**Fig. 13.**
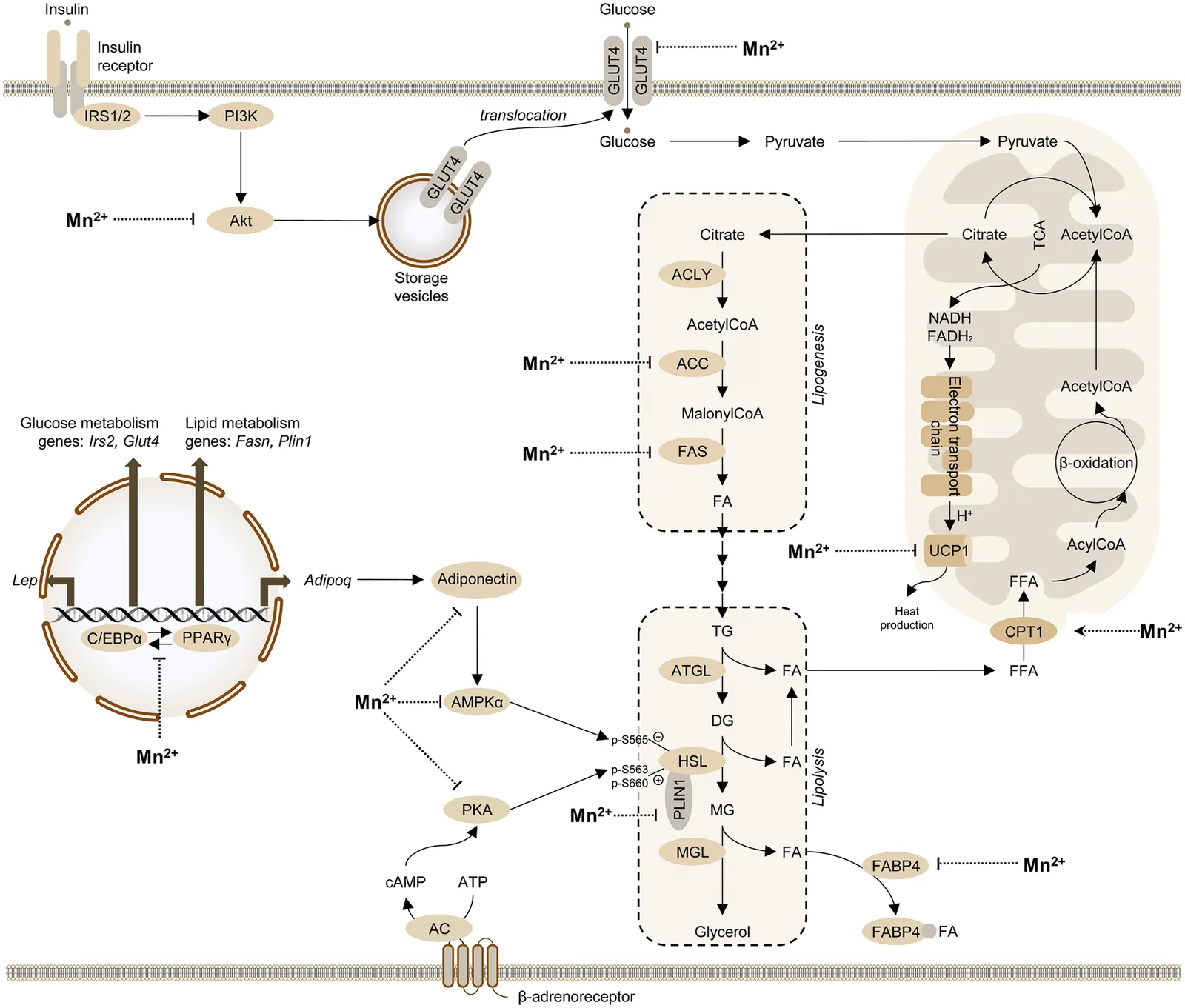
Summary of Early Mn exposure effects on fully differentiated adipocytes.

Given the global increase in Mn exposure from environmental and industrial sources, understanding how this metal influences adipose tissue development is critical for evaluating metabolic health risks. In summary, our novel findings indicate that transient Mn exposure during the early post-confluence window before hormonal induction was associated with persistent, concentration-dependent alterations in 3T3-L1 adipocyte differentiation and function, including lasting reductions in PPARγ and C/EBPα and downstream effectors, such as FABP4, leading to impaired lipid accumulation, and disrupts the endocrine profile, leading to a significant loss of their functional identity that does not require a sustained chronic exposure. These findings suggest that Mn exposure during a critical early stage of the differentiation protocol can produce enduring changes in adipocyte maturation without requiring sustained chronic exposure to Mn (Fig. 13).

Importantly, the interpretation of these effects must consider Mn concentration. Effects observed at lower and intermediate concentrations may reflect altered differentiation-associated programming, whereas the most pronounced effects observed at 500 μM likely include generalized metabolic impairment, cellular stress, and/or toxicity. Therefore, downstream reductions in adipogenic markers at the highest Mn concentration should not be attributed solely to specific changes in the adipogenic program. Further studies are needed to determine whether developmental Mn exposure affects adipocyte differentiation, energy balance, and obesity risk, including the potential role of impaired thermogenic function.

## Supplementary Material

1

Supplementary material associated with this article can be found, in the online version, at doi:10.1016/j.hazadv.2026.101506.

## Data Availability

Data will be made available on request.
