# Supplementary material for: Persistent effects of pre-differentiation manganese exposure on adipogenesis and metabolic function in differentiated 3T3-L1 adipocyte cells
Source: J Hazard Mater Adv. Author manuscript; Available in PMC 2026 Sep 22. (PMC13593052; doi:10.1016/j.hazadv.2026.101506)
Supplement: Supplementary file 1 [file NIHMS2210215-supplement-1.pdf]

SUPPLEMENTARY FIGURES

Supplementary figure 1.

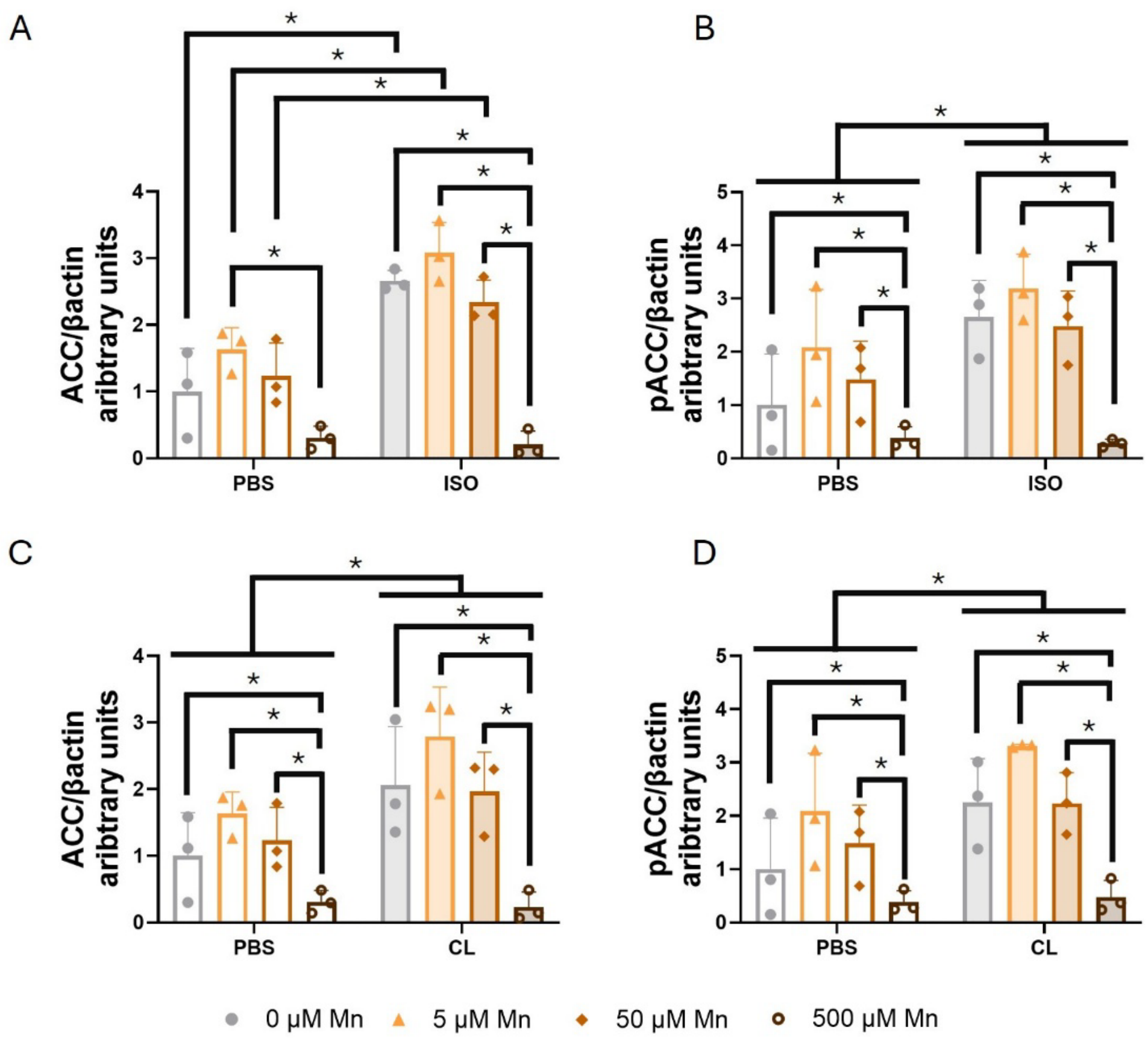

Supplementary figure 1. Transient Mn exposure during differentiation persistently impairs lipogenic program in fully differentiated adipocytes.

Transient Mn exposed (0, 5, 50, or 500 μM) mature adipocytes were treated with 10 μM of non-selective β-adrenergic receptor agonist Isoproterenol (ISO) or with 10 μM of β3-adrenergic receptor agonist CL 316,243 (CL) for 30 minutes in a humidified incubator at 37°C with 5% CO_2. Following treatment, Protein expression levels of lipogenic proteins were analyzed by western blot. **(A)** Total ACC quantified by densitometry in adipocytes treated with ISO. **(B)** Phospho-ACC quantified by densitometry in adipocytes treated with ISO. **(C)** Total ACC quantified by densitometry in adipocytes treated with CL. **(D)** Phospho-ACC quantified by densitometry in adipocytes treated with CL Data are presented as mean ± SD. Statistical significance was determined using two-way ANOVA followed by Bonferroni's post hoc test. $P < 0.05$ was

considered statistically significant. * indicates significant differences. Each point represents an independent biological replicate from a separate cell culture.

Supplementary figure 2.

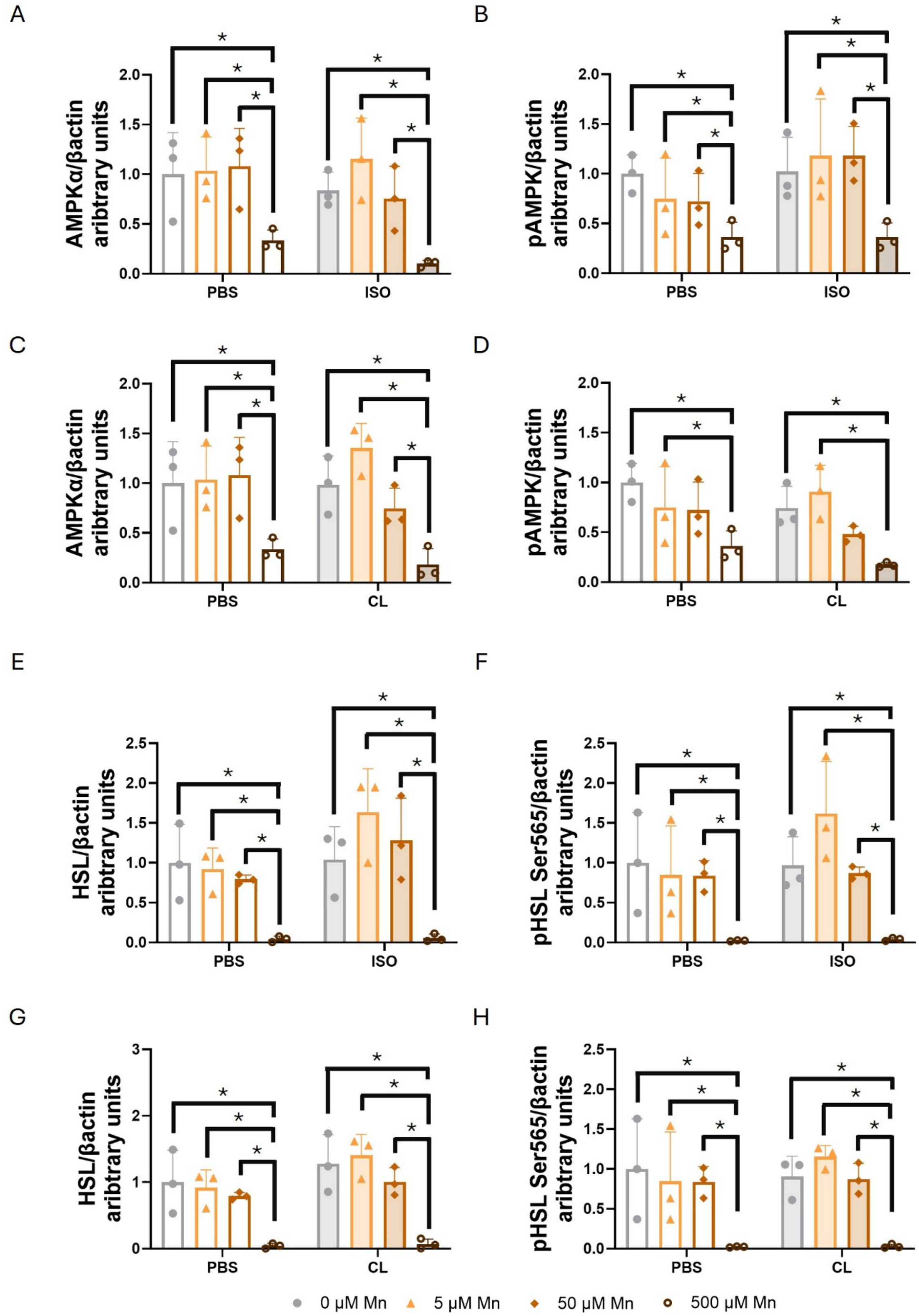

Supplementary figure 2. Early Mn exposure during differentiation persistently impairs β-adrenergic-AMPK-HSL signaling in fully differentiated adipocytes.

Transient Mn exposed (0, 5, 50, or 500 μM) mature adipocytes were treated with 10 μM of non-selective β-adrenergic receptor agonist Isoproterenol (ISO) or with 10 μM of β3-adrenergic receptor agonist CL 316,243 (CL) for 30 minutes in a humidified incubator at 37°C with 5% CO_2. Following treatment, protein expression levels of AMPK and AMPK-phosphorylated HSL were analyzed by western blot. **(A)** Total AMPKα quantified by densitometry in adipocytes treated with ISO. **(B)** Phospho-AMPKα quantified by densitometry in adipocytes treated with ISO. **(C)** Total AMPKα quantified by densitometry in adipocytes treated with CL. **(D)** Phospho-AMPKα quantified by densitometry in adipocytes treated with CL. **(E)** Total HSL quantified by densitometry in adipocytes treated with ISO. **(F)** phospho-HSL Ser565 quantified by densitometry in adipocytes treated with ISO. **(G)** Total HSL quantified by densitometry in adipocytes treated with CL. **(H)** Phospho-HSL Ser565 quantified by densitometry in adipocytes treated with CL. Data are presented as mean ± SD. Statistical significance was determined using two-way ANOVA followed by Bonferroni's post hoc test. $P < 0.05$ was considered statistically significant. * indicates significant differences. Each point represents an independent biological replicate from a separate cell culture.

Supplementary figure 3.

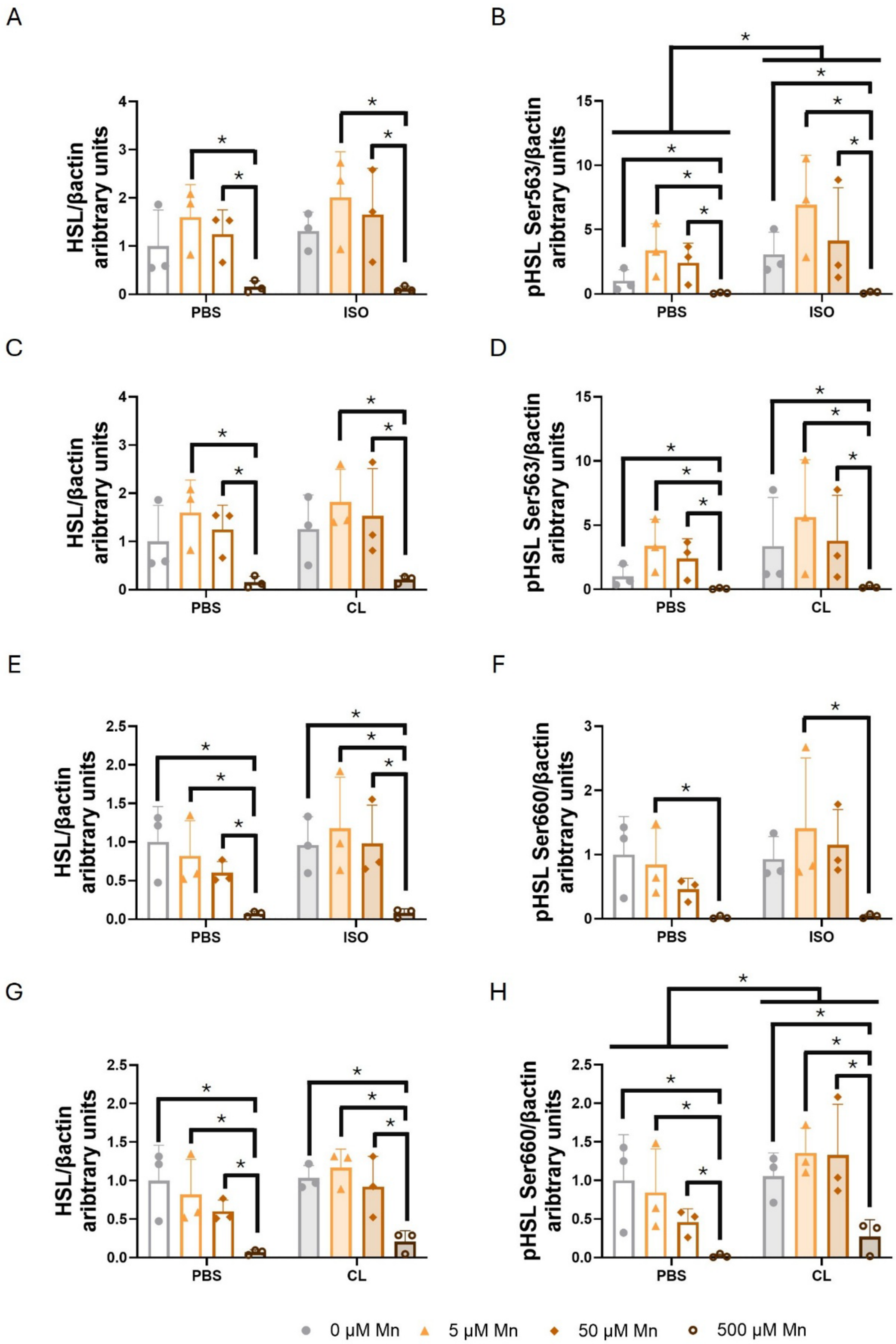

Supplementary figure 3. Early Mn exposure during differentiation persistently impairs β-adrenergic-HSL phosphorylation in fully differentiated adipocytes.

Transient Mn exposed (0, 5, 50, or 500 μM) mature adipocytes were treated with 10 μM of non-selective β-adrenergic receptor agonist Isoproterenol (ISO) or with 10 μM of β3-adrenergic receptor agonist CL 316,243 (CL) for 30 minutes in a humidified incubator at 37°C with 5% CO_2. Following treatment, protein phosphorylation of HSL was analyzed by western blot. **(A)** Total HSL quantified by densitometry in adipocytes treated with ISO. **(B)** Phospho-HSL Ser563 quantified by densitometry in adipocytes treated with ISO. **(C)** Total HSL quantified by densitometry in adipocytes treated with CL. **(D)** Phospho-HSL Ser563 quantified by densitometry in adipocytes treated with CL. **(E)** Total HSL quantified by densitometry in adipocytes treated with ISO. **(F)** Phospho-HSL Ser660 quantified by densitometry in adipocytes treated with ISO. **(G)** Total HSL quantified by densitometry in adipocytes treated with CL. **(H)** Phospho-HSL Ser660 quantified by densitometry in adipocytes treated with CL. Data are presented as mean ± SD. Statistical significance was determined using two-way ANOVA followed by Bonferroni's post hoc test. $P < 0.05$ was considered statistically significant. * indicates significant differences. Each point represents an independent biological replicate from a separate cell culture.
